# *In vitro* isolation of class-specific oligonucleotide-based small-molecule receptors

**DOI:** 10.1093/nar/gkz224

**Published:** 2019-03-30

**Authors:** Weijuan Yang, Haixiang Yu, Obtin Alkhamis, Yingzhu Liu, Juan Canoura, Fengfu Fu, Yi Xiao

**Affiliations:** 1 Department of Chemistry and Biochemistry, Florida International University, 11200 SW Eighth Street, Miami, FL 33199, USA; 2 Department of Plant Protection, Fujian Agriculture and Forestry University, Fuzhou 350002, China; 3 Key Laboratory for Analytical Science of Food Safety and Biology of MOE, Fujian Provincial Key Lab of Analysis and Detection for Food Safety, College of Chemistry, Fuzhou University, Fuzhou 350116, China

## Abstract

Class-specific bioreceptors are highly desirable for recognizing structurally similar small molecules, but the generation of such affinity elements has proven challenging. We here develop a novel ‘parallel-and-serial’ selection strategy for isolating class-specific oligonucleotide-based receptors (aptamers) *in vitro*. This strategy first entails parallel selection to selectively enrich cross-reactive binding sequences, followed by serial selection that enriches aptamers binding to a designated target family. As a demonstration, we isolate a class-specific DNA aptamer against a family of designer drugs known as synthetic cathinones. The aptamer binds to 12 diverse synthetic cathinones with nanomolar affinity and does not respond to 11 structurally similar non-target compounds, some of which differ from the cathinone targets by a single atom. This is the first account of an aptamer exhibiting a combination of broad target cross-reactivity, high affinity and remarkable specificity. Leveraging the qualities of this aptamer, instantaneous colorimetric detection of synthetic cathinones at nanomolar concentrations in biological samples is achieved. Our findings significantly expand the binding capabilities of aptamers as class-specific bioreceptors and further demonstrate the power of rationally designed selection strategies for isolating customized aptamers with desired binding profiles. We believe that our aptamer isolation approach can be broadly applied to isolate class-specific aptamers for various small molecule families.

## INTRODUCTION

It is highly advantageous to be able to sensitively detect multiple different members of a particular molecular family or class in many analytical contexts—for example, detecting illicit drugs and their metabolites for forensic investigations, antibiotics for food safety or pesticides for environmental monitoring (1–3). Cross-reactive assays that can broadly detect small molecules based on a shared molecular framework offer a more efficient and cost-effective solution to this problem than the tandem use of multiple highly specific assays that each detects an individual analyte. Antibody-based immunoassays have dominated the field of small-molecule detection (4), and while assays have been developed for a wide variety of individual targets, the development of class-specific immunoassays has proven difficult (5,6). This is in part because the process of antibody generation, which is performed *in vivo*, provides no control over the target-binding affinity and spectrum of the resulting antibody. Nucleic acid-based affinity reagents known as aptamers hold much promise in circumventing many of the shortcomings associated with antibodies (7). Aptamers are isolated through a process termed systematic evolution of ligands by exponential enrichment (SELEX) (8) to bind targets of interest with high affinity and specificity. Unlike antibodies, aptamers can be isolated relatively quickly and chemically synthesized in an inexpensive manner with no batch-to-batch variation. Moreover, aptamers are thermostable and have shelf lives of a few years at room temperature (9).

Theoretically, since SELEX is an *in vitro* process, the selection strategy and conditions can be precisely controlled to isolate class-specific aptamers that can broadly bind to small molecules sharing the same core structure. However, little work has been done to demonstrate the capability of SELEX to achieve such a goal. Aptamers isolated for a given small-molecule target often have innate cross-reactivity to analogs of that molecule, but the target-binding spectra of these aptamers are often either insufficient and/or unpredictable. For example, one heavily studied cocaine-binding aptamer (10) can also bind to metabolites such as norcocaine and cocaethylene, but does not respond to the major metabolite benzoylecognine, which only differs from cocaine by a single methyl group (11,12). Toggle-SELEX was developed as a solution to isolate cross-reactive aptamers (13). In this strategy, a library pool is challenged with two different targets sharing the same core structure, which are alternated every round to select for an aptamer that can cross-react to both targets—and ideally, target analogs sharing the same core structure. This method has led to the isolation of cross-reactive aptamers for a few structurally related small molecules (14–16). However, these aptamers typically exhibit only limited cross-reactivity (14,15), and the overall success rate of such approaches has been low (16). For example, Derbyshire *et al.* successfully isolated an aptamer that can bind to eight aminoglycoside antibiotics using Toggle-SELEX. Out of four independent selections with four different target pairs, only one yielded the final aptamer (16). The limitations of Toggle-SELEX could be attributed to two reasons. First, in previously reported approaches, only a fraction of possible substituent positions was varied between the two targets, yielding aptamers with narrow target-binding spectra. Second, since only a small portion of the aptamers in the initial pool are cross-reactive compared to those that bind to at least one target, those cross-reactive aptamers could be lost during Toggle-SELEX.

To overcome these problems, we have developed a new ‘parallel-and-serial’ selection strategy for SELEX to isolate class-specific aptamers for small molecule families. Our strategy has three steps that are crucial for its success. First, a set of structurally related targets are selected to define the core structure that is to be recognized by the isolated aptamer. The use of these targets creates selection pressure for aptamers that recognize generic molecular features common to all targets while remaining insensitive to peripheral substituents. Accordingly, it is important to choose as many targets as needed to represent variations at all desired substituent sites while preserving the core molecular framework of the target family. Next, these various targets are employed in a parallel selection process, in which multiple aptamer pools are enriched using each individual target. As a result, cross-reactive aptamers recognizing the shared core structure are enriched in all of the resulting pools after a few rounds of selection. When all these parallel pools are combined, the population of such aptamers should be relatively high. Finally, this combined pool is subjected to serial selection with each target sequentially, a process that ultimately retains only those aptamers that bind to the core structure shared by these targets. Importantly, this selection strategy is supplemented with a well-designed counter-SELEX procedure (17) to further define the targeted core structure and to prevent the aptamer from binding to structurally similar non-target molecules.

As a demonstration of this strategy, we isolated a class-specific aptamer for synthetic cathinones, a large family of dangerous designer drugs (18) that are associated with many severe psychological and physiological health consequences (19,20). The isolated aptamer demonstrated low nanomolar binding affinity to 12 synthetic cathinones while having no response to 11 structurally similar non-cathinone molecules. Analysis of the aptamer isolation process via high-throughput sequencing revealed that cross-reactive sequences were enriched during parallel selection, and that exponential enrichment of such aptamers occurred during serial selection. The target-binding spectrum of the aptamer was further evaluated via a dye-displacement sensing platform. Impressively, the aptamer enabled instantaneous colorimetric detection of several synthetic cathinones at nanomolar concentrations in biological samples, rivaling the performance of any currently available immunoassays that can only detect a few members of this family. To our knowledge, this is the first demonstration of using a rationally designed strategy to isolate high-affinity aptamers that are truly class-specific, having both broad target-binding spectrum as well as excellent specificity. Such success shows that the customizable nature of SELEX makes it well-suited for sourcing receptors with specific, yet broad molecular recognition capabilities, and that the approach described herein can be generally employed to directly isolate class-specific aptamers for any small-molecule family.

## MATERIALS AND METHODS

### SELEX strategy

The isolation of aptamers was carried out via a parallel-and-serial selection strategy. The whole aptamer isolation process consisted of five (for ethylone and butylone) or nine (for alpha-pyrrolidinopentiophenone, α-PVP) rounds of parallel selection and two cycles of serial selection. Detailed information regarding the conditions for each round of selection are listed in Supplementary Table S1. Since each sequence has a low copy number at the beginning of SELEX, high target concentrations were used during the first few rounds to ensure retention of all possible target binders. Moreover, for counter-SELEX, counter-target concentrations were initially kept low to remove high-affinity interferent binders while avoiding loss of target binders. In later rounds of SELEX, selection stringency was increased by decreasing the target concentration and increasing the counter-target concentration; this was done to obtain aptamers with high target affinity and specificity. The counter-SELEX protocol was rationally designed to encompass known and commonly observed interferents found in seized drug samples, including cutting agents (e.g. caffeine and acetaminophen), adulterants (e.g. procaine, lidocaine and promazine), other illicit drugs (e.g. cocaine and methamphetamine) or structurally related non-cathinone molecules (e.g. amphetamine, pseudoephedrine and ephedrine) to ensure that the isolated aptamer did not bind to them.

### Parallel selection

Parallel selection began with three initial pools consisting of 1 nmole DNA library (Supplementary Table S2) for each of the three different selection targets (α-PVP, ethylone and butylone). From Round P2 to P9, ∼300 pmole of enriched library pool for each target from the previous round was employed in the subsequent round. Positive selection was performed with progressively decreasing target concentrations (Round P1: 1 mM, Rounds P2–P3: 500 μM, Rounds P4–P5: 250 μM for α-PVP, ethylone and butylone; Rounds P6–P9: 100 μM for α-PVP only) to increase selection stringency for the enrichment of strong binders. For every round after the first, counter-SELEX was performed prior to positive selection in order to eliminate non-specific binders. The number and concentrations of counter-targets were progressively increased during the selection process to increase selection stringency. Specifically, from Rounds P2–P5 for the ethylone and butylone pools and Rounds P2–P8 for the α-PVP pool, we used the following counter-SELEX strategy: 100 μM cocaine for Round P2, a mixture of 100 μM cocaine and 100 μM procaine for Round P3, and a mixture of 100 μM cocaine, 100 μM procaine and 100 μM lidocaine for Rounds P4–P8. For Round P9 for the α-PVP pool, 300 μM each of cocaine, procaine and lidocaine was employed consecutively for counter-SELEX (Supplementary Table S1).

### Serial selection

We combined 100 pmole each from the P5 parallel pools for ethylone and butylone and the P9 parallel pool for α-PVP to generate the initial pool for serial selection. Two cycles of serial selection (Cycle 1 comprising Rounds S1–S3, Cycle 2 comprising Rounds S4–S6) were performed to specifically isolate cross-reactive aptamers. For each cycle, positive selection was performed by alternating the selection target (Rounds S1 and S4: butylone, Rounds S2 and S5: ethylone and Rounds S3 and S6: α-PVP). Target concentration was maintained at 100 μM for each round of serial selection for maximum stringency. In the first cycle of serial selection, counter-SELEX was performed prior to each round of positive selection by consecutively challenging the pool with 500 μM each of cocaine, procaine and lidocaine, while in the second cycle of serial selection, counter-SELEX was performed by sequentially challenging with a mixture of 500 μM each of ephedrine, pseudoephedrine, acetaminophen, methamphetamine and amphetamine; then with a mixture of 1 mM each of cocaine, procaine and lidocaine; and finally with 500 μM promazine (Supplementary Table S1).

### SELEX procedure

The isolation of aptamers was carried out following a previously reported library-immobilized SELEX protocol (21). The initial single-stranded DNA library used for SELEX consisted of approximately 6 × 10^14^ oligonucleotides. Each library strand is stem–loop structured and 73 nucleotides in length, with a randomized 30-nt loop flanked in turn by a pair of 8-nt stem-forming sequences and two primer-binding regions (Supplementary Table S2, DNA library). For each round of SELEX, the library/pool was mixed with biotinylated capture strands (Supplementary Table S2, cDNA-bio) at a molar ratio of 1:5 in selection buffer (10 mM Tris–HCl, 0.5 mM MgCl_2_, 20 mM NaCl, pH 7.4), heated at 95°C for 10 min and cooled at room temperature for over 30 min to ensure hybridization between library and capture strands. A micro-gravity column (0.5 ml) was prepared by adding 250 μl of streptavidin-coated agarose beads followed by three washes with 250 μl of selection buffer. About 250 μl of cDNA-library solution was then flowed through the micro-gravity column three times in order to conjugate the library to the agarose beads (Supplementary Figure S1A). The column was subsequently washed 10 times with selection buffer. Then, 250 μl of target (α-PVP, ethylone or butylone) dissolved in selection buffer was added to the column. Library molecules that bound to the target underwent a conformational change, which caused them to detach themselves from the biotinylated cDNA into solution (Supplementary Figure S1B). The eluent containing these strands was collected. This process was repeated twice, and all eluents were combined (750 μl total). The resulting pool was concentrated via centrifugation using a 3 kDa cut-off spin filter. The concentrated pool (100 μl) was then mixed with 1 ml of GoTaq Hot Start Colorless Master Mix with 1 μM forward primer (Supplementary Table S2, FP) and 1 μM biotinylated reverse primer (Supplementary Table S2, RP-bio) to amplify the pool via polymerase chain reaction (PCR). Amplification was performed using a BioRad C1000 thermal cycler with conditions as follows: 2 min at 95°C; 13 cycles of 95°C for 15 s, 58°C for 30 s and 72°C for 45 s, and 5 min at 72°C. The optimal number of amplification cycles was determined by performing pilot PCR to ensure sufficient amplification of enriched sequences without generating PCR-related artifacts. Amplification of the enriched pool and the absence of byproducts were confirmed using 3% agarose gel electrophoresis. If byproducts with differing lengths from the original library strands were observed, the pool was purified with a 4% agarose gel and the 73-nt products were recovered by silica column as reported previously (21). To generate single-stranded DNA from the resulting double-stranded PCR products, a fresh micro-gravity column was prepared containing 250 μl streptavidin-coated agarose beads, as described above. The amplified pool was then flowed through the column three times to conjugate the pool to the beads. Afterward, the column was washed six times with 250 μl of separation buffer (10 mM Tris–HCl, 20 mM NaCl, pH 7.4). The column was then capped, and 300 μl of a 0.2 M NaOH solution was added to the column and incubated for 10 min to generate single-stranded DNA, after which the eluent was collected. An additional 100 μl of 0.2 M NaOH was added to elute residual library strands from the column. Both eluents were combined and neutralized with 0.2 M HCl, and the pool was then concentrated via centrifugation with a 3 kDa cut-off spin filter. For every round after the first, counter-SELEX was performed before the positive selection step. Specifically, the library-immobilized column was washed with 250 μl of counter-target(s) in selection buffer to remove non-specific DNA strands. This process was performed three times for Rounds P1–P3 and S1–S6, and ten times for Rounds P4–P5 and, in the case of α-PVP, Rounds P4–P9. Afterward, the column was washed 30 times with selection buffer to wash away non-specific binders in preparation for positive selection.

### Gel elution assay

The enrichment, target affinity, specificity and cross-reactivity of the pools collected after Rounds P5 (for α-PVP, ethylone and butylone), P9 (for α-PVP only), S3 and S6 were evaluated using a modified version of a previously reported gel elution assay (Supplementary Figure S2) (22). Specifically, 50 pmole of enriched library (Supplementary Figure S2A) was incubated with 250 pmole of biotinylated cDNA in 125 μl of selection buffer, heated at 95°C for 10 min and cooled at room temperature over 30 min to anneal both strands and form cDNA-library complex (Supplementary Figure S2B). Afterward, a microcentrifugation column was prepared by adding 125 μl of streptavidin-coated agarose beads. The cDNA-library complex was then added to the column and immobilized on the beads (Supplementary Figure S2C), and the eluent was collected and recycled through the column twice more. The library-immobilized agarose beads were transferred into a microcentrifugation tube and washed five times by adding 625 μl of selection buffer, incubating on an end-over-end rotator for 5 min, followed by centrifugation and removal of the supernatant. The volume of the library-immobilized bead solution was adjusted to 150 μl with selection buffer and aliquoted into seven tubes (20 μl/tube). Afterward, 50 μl of target at a variety of final concentrations (0, 10, 50, 100, 250, 500 or 1000 μM) was added into each tube (Supplementary Figure S2D). After rotating for 60 min on an end-over-end rotator at room temperature, the beads were settled by centrifugation and 40 μl of the supernatant, which contained the target-eluted strands, was collected and set aside (Supplementary Figure S2E). Meanwhile, the leftover solution (30 μl) (Supplementary Figure S2F) was mixed with 50 μl of a 98% formamide solution containing 10 mM ethylenediaminetetraacetic acid and incubated at 90°C for 10 min to completely release all DNA strands from the beads (Supplementary Figure S2G). The resulting solution contained both leftover target-eluted strands and non-target-eluted strands. We analyzed the target-eluted aptamer solution and formamide-treated library solution via 15% denaturing polyacrylamide gel electrophoresis (PAGE) and determined the concentrations of the strands based on standardized concentrations of ladder loaded in the gel. The elution percentage was calculated using the equation:

\begin{equation*}\theta \ = {\rm{\ }}\frac{{{V_1} \times {c_{\rm s}}}}{{{V_2} \times {c_{\rm s}} + {\rm{\ }}{V_3} \times {c_{\rm b}}}} \times 100{\rm{\% }}\end{equation*}

where *θ* is the fraction of target-eluted strands, *c*_s_ is the concentration of target-eluted strands in the supernatant, *c*_b_ is the concentration of strands in the formamide solution, *V*_1_ is the volume of solution before supernatant collection (estimated as 62 μl, with ∼8 μl occupied by agarose beads), *V*_2_ is the volume of the collected supernatant containing target-eluted strands (40 μl) and *V*_3_ is the volume of solution after addition of formamide (80 μl). A calibration curve was created by plotting the fraction of eluted strands against the employed target concentration. The resulting curve was fitted with the Langmuir equation to determine the dissociation constant (*K*_D_) of the enriched pool. The same protocol was used to determine the target cross-reactivity and specificity of the enriched pool for other synthetic cathinones (4-MMC, 4-FMC, MDPBP, MDPV, MePBP, methedrone, methylone, MPHP, naphyrone, pentylone and pyrovalerone) or interferents (acetaminophen, amphetamine, caffeine, cocaine, ephedrine, lidocaine, methamphetamine, procaine, promazine, pseudoephedrine and sucrose).

### High-throughput sequencing (HTS) analysis of the aptamer isolation process

High-throughput sequencing (HTS) of the four final parallel pools and two serial pools was performed using Ion Torrent Sequencing. To prepare samples for sequencing, 10 nM pool was mixed with GoTaq Hot Start Colorless Master Mix, 1 μM forward primer and 1 μM reverse primer with a final volume of 50 μl. Nine cycles of PCR was then performed with the same PCR conditions as described in ‘SELEX Procedure’. Then 40 μl of PCR product was added into 16 μl of ExoSAP-IT reagent in an ice bath. The mixture was then incubated at 37 °C for 15 min to degrade remaining primers and nucleotides, followed by incubation at 80°C for 15 min to inactivate ExoSAP-IT reagent. HTS was performed at FIU DNA Core Facility using an Ion Personal Genome Machine System with an Ion 318 v2 chip (ThermoFisher Scientific). Upon obtaining the sequencing data, the primer sequences were trimmed by cutadapt (23), and the population of sequences from each pool were calculated using FASTAptamer (24). Members of the SCA2.1 family are defined as sequences containing ‘AGTGGGGTTCGGGTGGAGTT’ in any position in the 30-nt random region. The total reads for each pool are: ethylone P5: 234428, ethylone P8: 251376, butylone P5: 532967, a-PVP P9: 328474, S3: 297379, S5: 1575678.

## RESULTS

### Choosing targets for selection

**Figure 1.**
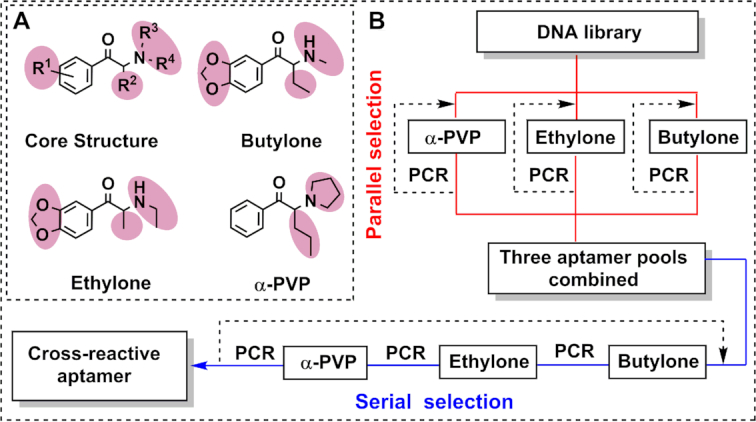
Isolation of a class-specific aptamer using a parallel-and-serial selection strategy. (**A**) The core structure of synthetic cathinones and the three targets chosen for parallel-and-serial selection, with substituent moieties shaded in red. (**B**) Schematic diagram of parallel-and-serial selection. Each of the three targets was subjected to parallel selection to enrich for broadly synthetic cathinone-specific aptamers (top), after which the resulting pools were combined and subjected to serial selection screening (bottom) to eliminate target-specific binders while retaining those aptamers with broad cross-reactivity for this drug class.

Synthetic cathinones share the same β-keto phenethylamine chemical core structure and typically differ at four substituent sites (Figure 1A, core structure). Performing selection directly against this ‘bare core’ (i.e. 2-amino-propiophenone, also known as cathinone) may yield aptamers with high affinity toward cathinone, but there is no guarantee that such aptamers will bind other synthetic cathinones that have different substituents on the core structure. This is supported by previous studies that showed that small molecule-binding aptamers isolated for a single target often have lower or no affinity for compounds with additional/differing substituents (17,25). We rationalized that performing SELEX with a set of structurally similar, yet sufficiently diverse, targets would create selection pressure for isolating aptamers that tolerate variations at all such sites, greatly increasing the likelihood that the isolated aptamer will have high cross-reactivity to this family as a whole. Thus, to isolate class-specific synthetic cathinone-binding aptamers, we selected three targets that share the same core structure but have variations at all of the substitution sites that are typically modified in this family: alpha-pyrrolidinovalerophenone (α-PVP), ethylone and butylone (Figure 1A).

### Parallel selection

Parallel selection was performed using three different initial library pools, with one pool being challenged with α-PVP, one with ethylone and one with butylone (Figure 1B, top). Presumably, challenging the parallel pools with individual targets enriches all sequences binding to the target, including those that are also cross-reactive to other synthetic cathinones. In contrast, challenging a single pool with a mixture of targets may lead to loss of cross-reactive aptamers. This is evidenced by a previous study that demonstrated that performing SELEX with a mixture of four targets yielded aptamers that specifically bind to just one of the four (26). This may be attributable to competitive binding among these targets.

To perform parallel selection, we employed a library pool consisting of ∼6 × 10^14^ unique oligonucleotide sequences, which form an 8-bp stem and a 30-nt random loop as the putative target-binding domain (Supplementary Figure S1A). During the first round, each initial library pool was challenged with 1 mM target, and eluted strands were collected and amplified for the next round of selection. To further establish class-specificity, from the second round onward we performed counter-SELEX prior to the positive selection step to remove aptamers binding to structurally similar interferents (e.g. cocaine, procaine, lidocaine) that have the same functional groups or partial structural features as our targets. In round two, counter-SELEX was first performed for each pool against 100 μM cocaine, followed by positive selection with 500 μM target, as reducing target concentration increases selection stringency (27–29). In the third round, a mixture of 100 μM cocaine and 100 μM procaine was used for counter-SELEX, with the same target concentration for positive selection. In rounds four and five, counter-SELEX was performed against cocaine, procaine and lidocaine (each at a concentration of 100 μM) in a mixture, with 250 μM target used for the positive selection step.

After the fifth round, a gel-elution assay (see ‘Materials and methods’ section) was performed to determine the target-binding affinity of each pool for its respective target. We observed that the fraction of eluted library increased with increasing target concentrations for the ethylone and butylone pools (Supplementary Figures S3A and S4A), showing that aptamers binding to these targets had been enriched through parallel selection. In contrast, target elution remained low for the α-PVP pool (Supplementary Figure S5A) regardless of the employed concentration of target, which indicated that the pool was not yet enriched. We further determined the cross-reactivity and specificity of the three pools via the gel-elution assay. The enriched ethylone and butylone pools were able to bind to both ethylone and butylone, but not to α-PVP, which indicated that the population of cross-reactive aptamers was relatively low. These pools also showed some affinity to procaine, with the ethylone pool also binding to cocaine. Neither pool displayed any affinity for lidocaine (Supplementary Figures S3B and S4B). In contrast, the α-PVP pool showed no affinity for any of the targets or counter-targets (Supplementary Figure S5B).

Given that the α-PVP pool was not yet enriched, we performed additional rounds of selection. From rounds six to eight, we used the same counter-target mixture of 100 μM cocaine, procaine and lidocaine from round 5 with a further-reduced α-PVP concentration of 100 μM for positive selection. For the ninth round, we used the same α-PVP concentration but with counter-selection performed with 300 μM of each of the three counter-targets in a consecutive manner. After the ninth round, we performed the gel-elution assay with the enriched pool and observed a clear target concentration-dependent elution profile for α-PVP, with an estimated dissociation constant (*K*_D_) of 28 μM (Supplementary Figure S6A). Notably, only 30% of the library was eluted, even in the presence of 1 mM α-PVP, which implied that there was just a small population of binders in the pool. We also determined that this pool displayed affinity to ethylone and butylone but was less responsive toward the various interferents (Supplementary Figure S6B), which can be attributed to the fact that more rounds of counter-SELEX were performed. Given that the pools enriched with individual targets also cross-reacted to other targets, it seemed likely that those pools contained cross-reactive aptamers. We believed that if parallel selection was continued, target-specific aptamers would have begun to dominate the pool; therefore, we terminated parallel selection before this could occur.

### Serial selection

**Figure 2.**
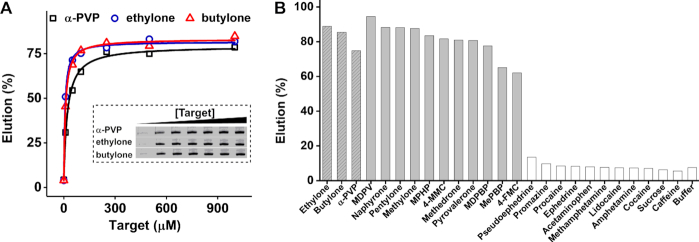
Characterization of the affinity and specificity of the final enriched pool via a gel-elution assay. (**A**) PAGE results depict the target elution profile, with lanes representing samples of the pool eluted with 0, 10, 50, 100, 250, 500 or 1000 μM (from left to right) α-PVP, ethylone or butylone. The percent of target-eluted pool was plotted against the concentration of target to determine the binding affinity of the enriched pool. (**B**) Percent elution values for 14 synthetic cathinones and 11 interferents at a concentration of 50 μM and buffer alone.

We then performed serial selection to enrich cross-reactive aptamers and exclude those specific to individual targets (Figure 1B, bottom). We combined all three enriched parallel pool as a starting library. For each cycle of serial selection, we challenged the combined pool with each target sequentially for a total of three rounds of selection using butylone (first round), ethylone (second round) and α-PVP (third round). In each round, we first performed counter-SELEX with 500 μM cocaine, procaine and lidocaine sequentially followed by positive selection with 100 μM target. After the first cycle of serial selection against all three targets, we performed the gel-elution assay to determine the cross-reactivity and specificity of the resulting pool. We observed that the cross-reactivity toward ethylone and butylone had substantially increased (*K*_D_ = 82 and 77 μM, respectively) relative to the individual pools obtained for these targets at the end of parallel selection, while affinity toward α-PVP was essentially unchanged (*K*_D_ = 34 μM) (Supplementary Figure S7A). Importantly, this pool exhibited greatly improved specificity, with minimal affinity for cocaine and lidocaine and only a moderate response to procaine (Supplementary Figure S7B). We then performed a second cycle of serial selection with an identical selection procedure but with a three-stage counter-SELEX process entailing sequential screening against a mixture of 500 μM each of ephedrine, pseudoephedrine, acetaminophen, methamphetamine, amphetamine, then a mixture of 1 mM each of cocaine, procaine and lidocaine, and finally with 500 μM promazine. We believe that the inclusion of these additional counter-targets, which are similar in structure to synthetic cathinones and commonly encountered in seized substances, further enhances the specificity of the enriched pool. After this cycle, we again evaluated the pool binding affinity via the gel-elution assay (Figure 2A). Each individual target (at a concentration of 500 μM) eluted more than 70% of the pool. The pool affinity toward ethylone and butylone had increased by ∼10-fold (*K*_D_ = 6.9 and 9.5 μM, respectively), whereas the affinity toward α-PVP only marginally increased (*K*_D_ = 21 μM).

### Characterization and sequencing of the final serial pool

We concluded that at this stage, the enriched pool largely comprised of cross-reactive synthetic cathinone-binding aptamers. To confirm this, we used the gel-elution assay to test the cross-reactivity of this pool by challenging it with the three targets as well as 11 other synthetic cathinones (for names and chemical structures, see Supplementary Figure S8A). All of them demonstrated >60% target elution at a concentration of 50 μM (Figure 2B). This shows that the aptamer could recognize the core structure of synthetic cathinones, while being tolerant even to side-chain substituents that were not encountered during SELEX. To evaluate the specificity of the enriched pool, we challenged the pool with 50 μM of the counter targets and other potential interferents (for chemical structures, see Supplementary Figure S8B) and found that none of them showed increased elution compared with buffer alone (Figure 2B). We therefore cloned and sequenced this final enriched pool. The pool was revealed to have low diversity, with 30 of the 50 clones having an identical sequence, which we named SCA2.1 (Supplementary Table S5). Mfold (30) predicts that SCA2.1 has a stem–loop structure with a 9-bp stem and a 28-nt loop in our selection buffer at room temperature (Supplementary Figure S9).

### High-throughput sequencing (HTS) analysis of the selection process

**Figure 3.**
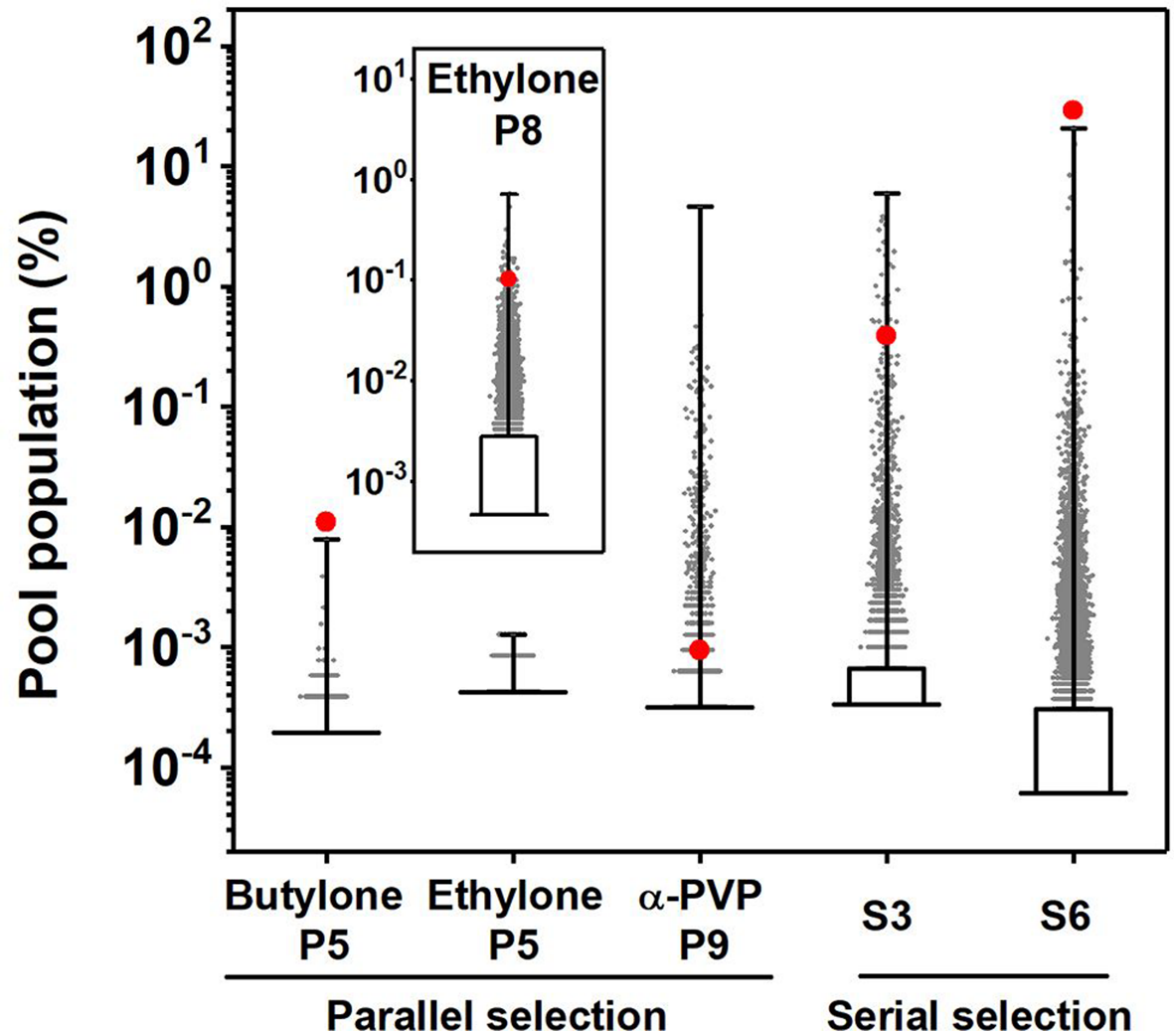
Box and whisker plots of the population distribution of sequences after parallel selection (butylone P5, ethylone P5, ethylone P8 and α-PVP P9) and each round of serial selection (S3 and S6) are shown. The longest horizontal line indicates the 50th percentile, with the boundaries of the box indicating the 5th and 95th percentile, and the whiskers indicate the highest and lowest values of the results. Sequences with population above 95th percentile are plotted as gray dots. The total population of SCA2.1 family is plotted as a red dot in each pool, except for the ethylone P5 pool where no such sequences were detected. Inset shows the pool population distribution after eight rounds of parallel selection using ethylone as target. Given the high diversity of the parallel pools, the lowest values 5th, 50th and 95th percentile all overlap, thus the box and lowest whisker cannot be seen. For the serial pools, the lowest values, 5th, and 50th percentile overlap, thus the bottom portion of the box and the lowest whisker are likewise not apparent.

We performed a thorough investigation of the parallel-and-serial selection process using HTS. Specifically, we sequenced our three final parallel pools (butylone P5, ethylone P5 and α-PVP P9) and two serial pools (S3 and S6) (Figure 3). Analysis of the parallel pools showed that the most prevalent sequence represented 0.0079% (butylone P5), 0.0012% (ethylone P5) and 4.6% (α-PVP P9) of their respective pool. The greater enrichment observed in the α-PVP P9 pool is probably due to the additional four rounds of parallel selection. Nevertheless, no particular sequence dominated any of these parallel pools, which indicated that they were not yet highly enriched. Meanwhile, the SCA2.1 family (see ‘Materials and methods’ section) comprised 0.011% of the butylone P5 pool and 0.00095% of the α-PVP P9 pool, which is notably higher than the median sequence abundance in each pool (0.00019% for butylone P5 and 0.00032% for α-PVP P9), showing that parallel selection enriches cross-reactive aptamers. We noted that ethylone P5 pool did not show any signs of enrichment. To determine if cross-reactive aptamers were present or lost in the ethylone pool, we performed an additional three rounds of parallel selection for this pool and then performed high-throughput sequencing with the resulting ethylone P8 pool. We found that the SCA2.1 family represented 0.1% of this pool, which implies that these sequences were originally present in the ethylone P5 pool, but at an amount below the detection capability of the HTS method we employed.

Upon combining the three parallel pools, the SCA2.1 family consisted of 0.0041% of the combined pool (median population: 0.000086%) based on our sequencing data. Such enrichment allowed for exponential enrichment of cross-reactive aptamers during serial selection, wherein the population of the SCA2.1 family increased to 0.39% of the S3 pool and 29% of the S6 pool. Indeed, parallel selection made serial selection highly efficient. It is likely that performing serial selection without parallel selection would result in loss of cross-reactive aptamers, given the generally low copy number of such sequences in initial rounds.

### Characterization of the affinity and specificity of the isolated aptamer

**Figure 4.**
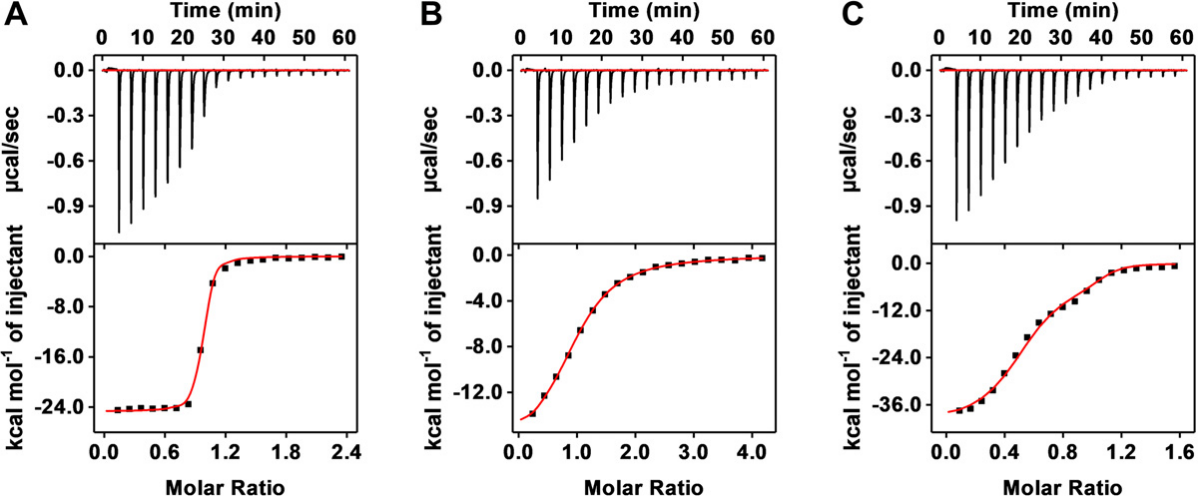
Characterization of the synthetic cathinone-binding affinity of SCA2.1 using ITC. Top panels present raw data showing the heat generated from each titration of (**A**) (−)-MDPV, (**B**) (+)-MDPV and (**C**) (±)-MDPV to SCA2.1, while bottom panels show the integrated heat of each titration after correcting for dilution heat of the titrant. ITC data obtained with (−)-MDPV and (+)-MDPV were fitted using a single-site model, and ITC data obtained with (±)-MDPV were fitted with a modified two-site binding model. All binding parameters are shown in Supplementary Table S4.

We then characterized the affinity of SCA2.1 for the selection targets and specificity against interferents using isothermal titration calorimetry (ITC). We titrated a 300–400 μM solution of target into a 20 μM solution of the aptamer, recorded the heat released by each titration and integrated these data to generate a binding curve. Curve fitting with a one-site binding model resulted in atypical binding stoichiometries (*N*) and less-than-optimal fitting, especially for the α-PVP titration curve that has a non-sigmoidal curve (Supplementary Figure S10 and Supplementary Table S3). Given that synthetic cathinones are chiral molecules and a racemic mixture of the targets was employed for SELEX, we hypothesized that the aptamer may have differential binding affinity for each enantiomer. The pure enantiomers of the three selection targets were not commercially available, but the high cross-reactivity of the aptamer allowed us to use enantiomers for another synthetic cathinone, (−)- and (+)-MDPV, to confirm our hypothesis. ITC data indicated that the aptamer binds to one target molecule, with *N* = 0.92 and 0.95 for (−)*-* and (+)-MDPV, respectively, and exhibits 100-fold greater affinity for the (−) enantiomer (*K*_D_ = 46.5 ± 7.5 nM) relative to the (+) enantiomer (*K*_D_ = 3.61 ± 0.12 μM) (Figures 4A and B and Supplementary Table S4). We also performed ITC by titrating racemic MDPV into a solution of SCA2.1 and observed a binding curve similar in appearance to that of α-PVP. Using a modified two-set-of-sites model (see ‘Supplementary materials and methods’ for detail) to fit the result (Figure 4C), we obtained similar binding parameters to those produced by the titration of each enantiomer alone (Supplementary Table S4, (±)-MDPV). This confirmed that the modified model is appropriate to describe such binding phenomenon.

**Figure 5.**
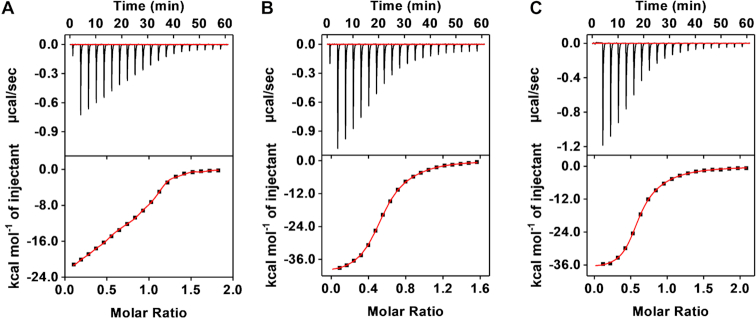
Characterization of the target-binding affinity of SCA2.1 using ITC. Top panels present raw data showing the heat generated from each titration of (**A**) α-PVP, (**B**) ethylone and (**C**) butylone to SCA2.1, while bottom panels show the integrated heat of each titration after correcting for dilution heat of the titrant. ITC data were fitted with a modified two-site binding model and the binding parameters are shown in Supplementary Table S3.

We then used this modified two-set-of-sites model to fit the ethylone, butylone and α-PVP binding curves, and obtained improved fitting and normal *N* values (0.9–1.1), with nanomolar affinity toward one enantiomer and micromolar affinity for the other (Figure 5 and Supplementary Table S3). Opposing intuition, these results suggest that SCA2.1 can achieve not only high target cross-reactivity, but also superior binding affinity. To determine the specificity of SCA2.1, we performed ITC with interferents that are most structurally similar to synthetic cathinones including amphetamine, methamphetamine, ephedrine, cocaine and procaine. All such compounds had very low binding affinity for SCA2.1 (Supplementary Figure S11).

### Evaluating the target-binding spectrum and specificity of the isolated aptamer

We then demonstrated the analytical utility of SCA2.1 in a colorimetric dye-displacement assay. Diethylthiotricarbocyanine (Cy7) is a small-molecule dye that exists in equilibrium between monomer and dimer forms, which have absorbance peaks at 760 and 670 nm, respectively. Previous studies have shown that Cy7 monomers can bind to hydrophobic target-binding domains of aptamers, which results in strong enhancement of absorbance at 760 nm (22,31). However, the binding of target to the aptamer can displace Cy7 monomer from the binding domain within seconds, which causes the dye to dimerize in aqueous solution, resulting in the reduction of absorbance at 760 nm and enhancement of absorbance at 670 nm (Supplementary Figure S12). This approach can thus be used as a colorimetric indicator for small molecule detection. We first examined if such an assay can be employed to detect synthetic cathinones using SCA2.1. We determined the binding affinity of Cy7 to SCA2.1 by titrating different concentrations of the aptamer into a solution of 2 μM Cy7 (Supplementary Figure S13A). Increasing the amount of aptamer progressively enhanced the absorbance of Cy7 monomer at ∼760 nm, indicating binding to the aptamer. A gradual peak shift from 760 to 775 nm was also observed, which is consistent with previous studies (22,31) showing that absorbance of the monomer can change in different microenvironments, such as when the dye binds to the aptamer. Based on Cy7 absorbance at 775 nm, we obtained a *K*_D_ of 1.6 μM (Supplementary Figure S13B). We then investigated whether the synthetic cathinone targets can efficiently displace Cy7 from SCA2.1. We first titrated different concentrations of butylone into a mixture of 2 μM Cy7 and 3 μM SCA2.1, and found that increasing concentrations of butylone progressively reduced the absorbance of Cy7 at 775 nm while enhancing absorbance at 670 nm (Supplementary Figure S14A). This change can be attributed to dimerization of the Cy7 monomer when displaced from the aptamer into solution (22,31). We used the absorbance ratio between 670 and 775 nm (*A*_670_/*A*_775_) to calculate signal gain and generate a calibration curve, which displayed a linear range of 0 to 10 μM and a measurable detection limit of 250 nM (Supplementary Figure S15). We obtained equivalent results with both ethylone and α-PVP (Supplementary Figures S14B,C and S15), again confirming the high cross-reactivity of SCA2.1. The Cy7-displacement assay is also compatible with biosamples such as urine and saliva, since the absorbance range of Cy7 is well outside the background absorbance exhibited by these matrices (22). We obtained calibration curves with ethylone spiked into 50% urine (Supplementary Figure S16) and 50% saliva (Supplementary Figure S17) with a linear range of 0 to 10 μM and a measurable detection limit of 80 and 120 nM, respectively. The enhanced sensitivity of the assay in these biomatrices can be possibly attributed to the higher ionic strength of the media, which may enhance target-binding to the aptamer or Cy7 dimerization.

**Figure 6.**
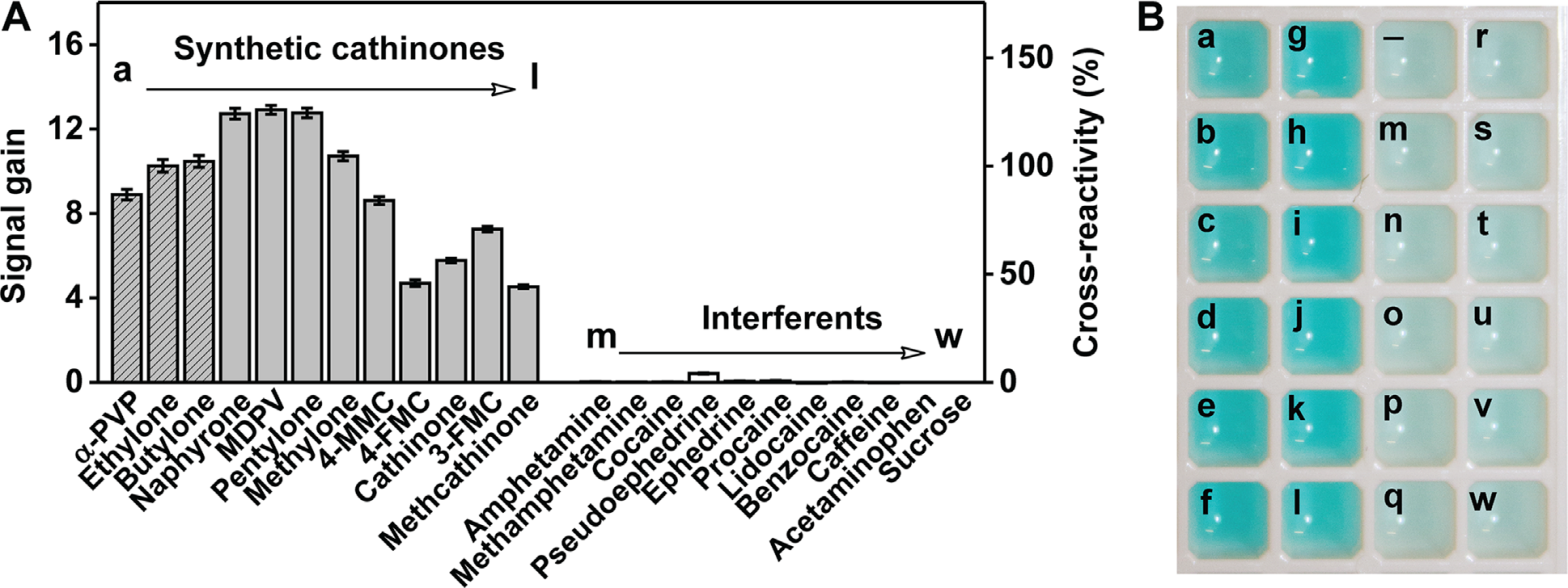
Colorimetric detection of synthetic cathinones using a SCA2.1-based Cy7-displacement assay. (**A**) Signal gain measured via a plate reader from the Cy7-displacement assay with 12 synthetic cathinones (gray, with the three selection targets shaded) and 11 interferents (white) at a concentration of 50 μM with 3 μM SCA2.1 and 2 μM Cy7. Cross-reactivity is defined as the ratio of signal gain between the reference target ethylone and another synthetic cathinone or interferent multiplied by 100%. Error bars show standard deviations from three measurements. (**B**) Naked-eye detection of synthetic cathinones using a mixture of 5 μM SCA2.1 and 3.5 μM Cy7. The color of the solution changes to bright blue within seconds upon addition of 50 μM synthetic cathinones (a-l). However, the color appears as a faint blue color in the absence of target (-) or 50 μM of a wide range of interferents (m-w).

We tested the cross-reactivity of this assay for nine other synthetic cathinones, including naphyrone, MDPV, pentylone, methylone, 4-MMC, 4-FMC, 3-FMC, methcathinone and cathinone at a concentration of 50 μM. As expected, despite the diversity of the side chains substituents, all synthetic cathinones induced a significant change in *A*_670_/*A*_775_, producing a signal gain ranging from 45% to 130% relative to ethylone (Figure 6A). This implies that SCA2.1 mainly recognizes the β-keto phenethylamine core structure, and variations in the side chains do not significantly affect target-binding affinity. Notably, the aptamer is more cross-reactive to a broad range of synthetic cathinones than antibodies used in existing immunoassays, which achieve >20% cross-reactivity for only five synthetic cathinones (32). SCA2.1 shows a moderate bias toward bulkier synthetic cathinones such as MDPV, naphyrone and pentylone. Such ligands may fit better in the binding pocket compared to smaller synthetic cathinones like methcathinone or 4-FMC, achieving high binding affinity through greater interaction with the aptamer. This supports the higher signal gain observed in the assay. Importantly, our assay has excellent specificity, as the aptamer does not cross-react to non-synthetic cathinone interferents. We tested our assay with 11 different interferent compounds, including common illicit drugs (amphetamine, methamphetamine and cocaine) and cutting agents found in street samples (pseudoephedrine, ephedrine, procaine, lidocaine, benzocaine, caffeine, acetaminophen and sucrose) at a concentration of 50 μM. The assay yielded no response to any of these interferents (Figure 6A), even though many contained a partial β-keto phenethylamine structure, demonstrating that aptamer specificity can be precisely controlled through a well-designed selection approach.

We finally determined the limit of detection for our Cy7-displacement assay for 12 synthetic cathinones and observed detection limits that varied from 40 to 80 nM in 50% urine (Supplementary Figure S18). Clearly, the high affinity of this aptamer as well as its unresponsiveness toward endogenous compounds enables sensitive screening of synthetic cathinones in biological samples. Given that the concentration of these drugs in urine typically ranges between high nanomolar to <100 μM within a few hours after consumption (33), we believe that our assay will be useful for label-free detection of synthetic cathinones in these matrices.

We further fine-tuned our Cy7-displacement assay by using a higher concentration of the dye and aptamer in order to intensify the target-induced color change and thereby enable naked-eye detection. We challenged this assay with the aforementioned 12 synthetic cathinones and 11 interferent compounds at a concentration of 50 μM with 3.5 μM Cy7 and 5 μM SCA2.1. In the absence of target, the aptamer-bound Cy7 monomer has an absorption peak at 775 nm, which is not in the visible range, and thus the sample is practically colorless. However, when Cy7 is displaced by the target, the resulting dimerization-associated absorption peak at 670 nm causes the solution to produce a bright, clearly visible blue color. We observed that all 12 synthetic cathinones immediately induced a clear-to-blue color change in the solution, while no color change was identified upon addition of any of the interferent compounds (Figure 6B). Using ethylone as a target, we determined that 6.3 μM is the lowest concentration that can develop a color distinguishable from the blank with the naked eye (Supplementary Figure S19). These results demonstrate the feasibility of the Cy7-displacement assay for instrument-free on-site drug screening applications.

## DISCUSSION

Class-specificity implies a degree of both receptor promiscuity toward targets within a designated molecular family and specificity against molecules outside of the family. The development of class-specific antibodies and aptamers has proven challenging due to the lack of viable methods to precisely control receptor binding profiles and specificity. In this work, we sought to develop a new aptamer isolation strategy, parallel-and-serial selection, as an effective way to select for class-specific aptamers recognizing small molecules based on a familial molecular core structure. The parallel-and-serial selection strategy increases the likelihood of isolating broadly cross-reactive aptamers by enriching oligonucleotide pools in parallel against diversely structured members of the designated target family and then combining and challenging the pools serially with these targets to fine tune specificity toward a particular class of targets. Here, we chose three synthetic cathinones that vary at all major substituent sites of the targeted family to ensure that broadly cross-reactive aptamers are isolated. Other target triplets could yield equally cross-reactive aptamers if they are sufficiently diverse. More targets can be employed to create broader cross-reactivity, although this will increase labor and cost requirements. By supplementing our strategy with counter-SELEX, the binding spectrum can be narrowed down to the target family, thereby avoiding unwanted cross-reactivity to structurally similar non-target molecules.

As a demonstration, we isolated a single class-specific DNA aptamer that can bind to 12 diverse synthetic cathinones. This aptamer is insensitive to variations at all substituent sites on the core structure, and even tolerates many substituents that do not appear in our selection targets. Importantly, our aptamer does not respond to 11 structurally similar compounds, some of which only differ from our targets by a single atom. We subsequently demonstrated the superior class-specificity and affinity of our aptamer in a single-step, colorimetric Cy7-displacement assay, which can detect clinically relevant concentrations of synthetic cathinones in biomatrices (33–35) and presents greater target cross-reactivity than existing antibodies. Advantageously, this assay can also achieve naked-eye detection of synthetic cathinones at concentrations at low micromolar concentrations, which is valuable for on-site screening of seized substances.

The aptamer isolated herein displays binding characteristics that confound intuition. This aptamer has great molecular promiscuity, binding to several molecules sharing a common, defined core structure. All the while, the aptamer retains the ability to discriminate against non-target molecules that are closely related in structure to members of the designated target family, even by a single atom difference in certain instances. Impressively, the aptamer also can bind to its targets with nanomolar affinity. This was not as expected, as broad cross-reactivity and high affinity is, on the surface, counter-intuitive.

Our findings significantly expand the capability of aptamers as class-specific biorecognition elements and demonstrate an unprecedented level of control over aptamer binding profiles through this new parallel-and-serial selection strategy. We believe that our approach can be used to isolate class-specific aptamers for other families of small molecules for applications relevant to medical diagnostics, environmental monitoring, food safety and forensic science.

## Supplementary Material

gkz224_Supplemental_File
