# Supplementary material for: In vitro isolation of class-specific oligonucleotide-based small-molecule receptors
Source: Nucleic Acids Res. 2019 Mar 30;47(12):e71. doi: 10.1093/nar/gkz224 (PMC6614805; doi:10.1093/nar/gkz224)
Supplement: gkz224_Supplemental_File [file gkz224_supplemental_file.pdf]

Supplementary data for the article:

In Vitro Isolation of Class-Specific Oligonucleotide-Based Small-Molecule Receptors

Weijuan Yang[1, 2†], Haixiang Yu[1†], Obtin Alkhamis[1], Yingzhu Liu[1], Juan Canoura[1], Fengfu Fu[3] and Yi Xiao[1*]

[1]Department of Chemistry and Biochemistry, Florida International University, 11200 SW Eighth Street, Miami, Florida 33199, United States; [2]Department of Plant Protection, Fujian Agriculture and Forestry University, Fuzhou, China, 350002; [3]Key Laboratory for Analytical Science of Food Safety and Biology of MOE, Fujian Provincial Key Lab of Analysis and Detection for Food Safety, College of Chemistry, Fuzhou University, Fuzhou, China, 350116.

†These authors contributed equally to this work.
*Corresponding author: yxiao2@fiu.edu

Reagents and materials. All DNA oligonucleotides were purchased from Integrated DNA Technologies (HPLC-purified) and dissolved in PCR water. The names and sequences of the DNA oligonucleotides are listed in **Supplementary Table S2**. The concentrations of dissolved DNA were measured using a NanoDrop 2000 spectrophotometer (Thermo Fisher Scientific). Synthetic cathinone standards (all hydrochloride salts and racemic unless specified), including 3,4-methylenedioxy-α-pyrrolidinobutiophenone (MDPBP), 3,4-methylenedioxypyrovalerone (MDPV), 3-fluoromethcathinone (3-FMC), 4-fluoromethcathinone (4-FMC), 4-methyl-α-pyrrolidinobutiophenone (MePBP), 4'-methyl-α-pyrrolidinohexiophenone (MPHP), 4-methylmethcathinone (4-MMC), alpha-pyrrolidinopentiophenone (α-PVP), butylone, cathinone, ethylone, methcathinone, methedrone, methylone, naphyrone, pentylone, pyrovalerone, and pure enantiomers (-)-MDPV and (+)-MDPV were purchased from Cayman Chemicals. Acetaminophen, (±)-amphetamine hemisulfate, benzocaine, caffeine, cocaine HCl, diethylthiatricarbocyanine iodide (Cy7), (-)-ephedrine HCl, lidocaine HCl, (+)-methamphetamine HCl, procaine HCl, promazine HCl, (+)-pseudoephedrine HCl, sucrose and all other chemicals were purchased from Sigma-Aldrich unless otherwise noted. Tween 20, formamide, SYBR Gold, streptavidin-coated agarose resin (capacity: 1-3 mg biotinylated BSA/ml resin), One Shot Chemically Competent *E. coli*, TOPO TA cloning kit, PureLink Quick Plasmid Miniprep Kit, and ExoSAP-IT Express PCR Purification Kit were purchased from ThermoFisher Scientific. 500 µL micro-gravity columns were purchased from Bio-Rad. GoTaq Hot Start Colorless Master Mix was purchased from Promega. 3 kDa cut-off spin filters were purchased from Millipore.

Characterization of Cy7 binding to SCA2.1. 8 µL of different concentrations of SCA2.1, 8 µL of 20 µM Cy7, and 64 µL of reaction buffer (final concentration 10 mM Tris-HCl, 0.5 mM $MgCl_2$, 20 mM NaCl, 0.01% Tween 20, and 1% DMSO, pH 7.4) were mixed in the wells of a transparent flat-bottomed 384-well plate. UV-vis spectra were immediately recorded from 450–900 nm using a Tecan Infinite M1000 PRO microplate reader at room temperature. The absorbance value at 775 nm (λ_{max} of Cy7 monomer) was plotted against the concentration of added aptamer. K_D was estimated by non-linear fitting using the Langmuir equation.

Cy7-displacement assay for colorimetric detection of synthetic cathinones. 8 µL of SCA2.1 (final concentration: 3 µM), 8 µL of Cy7 (final concentration 2 µM), 8 µL of varying concentrations of target (α-PVP, butylone, or ethylone), and 56 µL of reaction buffer (final concentration 10 mM Tris-HCl, 0.5 mM $MgCl_2$, 20 mM NaCl, 0.01% Tween 20, 1% DMSO, pH 7.4) were mixed in the wells of a transparent flat-bottomed 384-well plate. UV-vis spectra were immediately recorded from 450–900 nm using a Tecan microplate reader at room temperature. The absorbance ratio between 670 nm and 775 nm (A_{670}/A_{775}) was calculated for each sample, and signal gain was calculated by $(R–R_0)/R_0$, where R_0 and R represent A_{670}/A_{775} without and with target, respectively. Using the same protocol, the cross-reactivity and specificity of the assay were tested with other synthetic cathinones (naphyrone, MDPV, pentylone, methylone, 4-MMC, 4-FMC, 3-FMC, methcathinone and cathinone) and interferents (amphetamine, methamphetamine, cocaine, pseudoephedrine, ephedrine, procaine, lidocaine, benzocaine, caffeine, acetaminophen, and sucrose) at a concentration of 50 µM. Cross-reactivity was calculated using the signal gain of 50 µM ethylone as 100%. For visual synthetic cathinone detection, the assay was performed using the same protocol but with higher concentrations of SCA2.1 (5 µM) and Cy7 (3.5 µM) with 50 µM of the aformentioned synthetic cathinones or interferents. Samples were loaded into a white flat-bottomed 384-well plate. Photographs of the samples were taken using a digital camera immediately after mixing all reaction components.

Cloning and sequencing. Cloning and sequencing of the final enriched pool was performed using a previously reported protocol (1). Briefly, the enriched sequences from the final pool (Round S6) were amplified by PCR with unlabeled forward and reverse primers (**Supplementary Table S2, FP and RP**) using the same program as described above in 'SELEX procedure'. At the end of the amplification protocol, an additional 30 min extension step was performed at 72 °C to add a poly-A tail. Amplicons were cloned into a plasmid vector and transformed into *E. coli* cells using the TOPO TA cloning kit (Invitrogen) according to the supplier recommendations. The plasmids from 50 randomly picked colonies were extracted using a PureLink Quick Plasmid Miniprep Kit (Invitrogen) and sequenced via Sanger Sequencing at the Florida International University DNA Core Facility. After removing the sequences of the plasmid and primers, the aptamer sequences were aligned with BioEdit software to determine the consensus sequence.

Isothermal titration calorimetry (ITC). ITC was performed using a MicroCal ITC200 instrument (Malvern). All ITC experiments were carried out using the following protocol at 23 °C. A solution containing the aptamer (final concentration 20 µM) was prepared in selection buffer (10 mM Tris-HCl, 0.5 mM $MgCl_2$, 20 mM NaCl, pH 7.4) and loaded into the ITC sample cell. We then loaded different concentrations of synthetic cathinone (350 µM (±)-α-PVP, 300 µM (±)-ethylone, 400 µM (±)-butylone, 300 µM (±)-MDPV, 225 µM (-)-MDPV, or 400 µM (+)-MDPV) or interferent (1 mM (±)-amphetamine, (+)-methamphetamine, cocaine, procaine, or (+)-ephedrine) in the same buffer into the syringe and titrated it into the cell, with an initial 0.4 µL purge injection followed by 19 successive 2 µL injections. A spacing of 180 seconds was used between each titration. The heat generated from each titration was recorded, and the binding stoichiometry (N), enthalpy (ΔH), and K_D were obtained by fitting the resulting titration curve with a single-site binding model (wherein the aptamer and synthetic cathinone are considered to be the receptor and ligand, respectively) using the MicroCal analysis kit integrated into Origin 7 software. All titrations involving racemic synthetic cathinone mixtures were also fitted with a modified two-site binding model to determine the binding parameters for each enantiomer. This model assumes that a receptor with two ligand-binding sites independently interacts with a ligand. Unlike the single-site binding model, the aptamer in the sample cell is considered as the ligand and the pair of synthetic cathinone enantiomers is considered as a receptor with two discrete binding sites. For example, titration of 300 µM (±)-MDPV – containing 150 µM each of (-)-MDPV and (+)-MDPV – into 20 µM SCA2.1 is treated as titration of 150 µM of a receptor with two independent 'binding sites' into 20 µM of ligand. The binding parameters of the first site (*i.e.* N_1, K_{D1}, ΔH_1) reflect the binding of one of the enantiomers to the aptamer, while those for the second site (*i.e.* N_2, K_{D2}, ΔH_2) reflect the binding of the other.

Reference:

(1) Yang,K.A., Pei,R. and Stojanovic,M.N. (2016) In vitro selection and amplification protocols for isolation of aptameric sensors for small molecules. *Methods*, **106**, 58–65.

Table S1. Detailed information regarding the conditions for each round of SELEX.

	Round #	Pool size (pmole)	Counter-targets			Targets
Parallel selection	P1	1000	N/A			Butylone (1000 μM)
		1000				Ethylone (1000 μM)
		1000				α-PVP (1000 μM)
	P2	358	COC (100 μM)			Butylone (500 μM)
		346				Ethylone (500 μM)
		368				α-PVP (500 μM)
	P3	321	Mixture of COC & PRC (100 μM each)			Butylone (500 μM)
		362				Ethylone (500 μM)
		345				α-PVP (500 μM)
	P4	319	Mixture of COC, PRC, & LDC (100 μM each)			Butylone (250 μM)
		367				Ethylone (250 μM)
		292				α-PVP (250 μM)
	P5	300	Mixture of COC, PRC, & LDC (100 μM each)			Butylone (250 μM)
		300				Ethylone (250 μM)
		300				α-PVP (250 μM)
	P6	300	Mixture of COC, PRC, & LDC (100 μM each)			α-PVP (100 μM)
	P7	300	Mixture of COC, PRC, & LDC (100 μM each)			α-PVP (100 μM)
	P8	300	Mixture of COC, PRC, & LDC (100 μM each)			α-PVP (100 μM)
	P9	300	COC (300 μM)	PRC (300 μM)	LDC (300 μM)	α-PVP (100 μM)
Serial selection	S1	300*	COC (500 μM)	PRC (500 μM)	LDC (500 μM)	Butylone (100 μM)
	S2	300				Ethylone (100 μM)
	S3	300				α-PVP (100 μM)
	S4	300	Mixture of EPH, PSE, ACM, METH, & AMP (500 μM each)	Mixture of COC, PRC, & LDC (1000 μM each)	Promazine (500 μM)	Butylone (100 μM)
	S5	300				Ethylone (100 μM)
	S6	300				α-PVP (100 μM)

* consists of 100 pmole each of pool enriched with butylone (Round P5), ethylone (Round P5) and α-PVP (Round P9).

Acetaminophen (ACM), amphetamine (AMP), cocaine (COC), ephedrine (EPH), lidocaine (LDC), methamphetamine (METH), procaine (PRC), Pseudoephedrine (PSE).

Table S2. Sequences of DNA oligonucleotides employed in this work.

**Sequence ID**	**Sequence (5' – 3')**
DNA Library	CGAGCATAGGCAGAACTTACGAC(N30)<br>GTCGTAAGAGCGAGTCATTC
cDNA-bio	TTTTTGTCGTAAGTTCTGCCATTTT/Bio/
FP	CGAGCATAGGCAGAACTTAC
RP-bio	/Bio/GAATGACTCGCTCTTACGAC
RP	GAATGACTCGCTCTTACGAC
SCA2.1	CTTACGACCTTAAGTGGGGTTCGGGTGGAGTTTATGGGGTCGTAAG

N represents random base; /Bio/ represents biotin modification

Table S3. Binding parameters of SCA2.1 to α-PVP, ethylone and butylone, as characterized by fitting ITC data with a one-site or modified two-site binding model.

**Targets**	**α-PVP**		**Ethylone**		**Butylone**	
**Binding model**	**One-site**	**Two-site**	**One-site**	**Two-site**	**One-site**	**Two-site**
N_1 (target/aptamer)	1.66±0.05	1.10±0.01	1.09±0.01	1.06±0.06	1.20±0.01	1.06±0.09
**K_{D1} / μM**	2.36±0.55	0.09±0.02	1.62±0.06	0.10±0.03	1.81±0.13	0.16±0.05
ΔH_1 / kcal mol^{-1}	-10.8±0.5	-16.5±0.1	-21.5±0.1	-23.1±0.2	-19.9±0.3	-25.9±0.6
N_2 (target/aptamer)		0.91±0.16		0.90±0.03		0.98±0.04
**K_{D2} / μM**		18.5±7.7		2.22±0.09		1.59±0.14
ΔH_2 / kcal mol^{-1}		-12.2±4.4		-19.2±0.6		-12.9±2.7

Table S4. Binding parameters of SCA2.1 to (-)-MDPV, (+)-MDPV, (±)-MDPV, as characterized by fitting ITC data with a one-site (for (-)- or (+)-MDPV) or modified two-site binding model (for (±)-MDPV).

**Binding parameters**	**(-)-MDPV**	**(+)-MDPV**	**(±)-MDPV**
N_1 (target/aptamer)	0.92±0.01		1.04±0.06
**K_{D1} / nM**	46.5±7.5		17.5±16.4
ΔH_1 / kcal mol^{-1}	-24.7±0.2		-22.3±0.4
N_2 (target/aptamer)		0.95±0.01	0.91±0.08
**K_{D2} / μM**		3.61±0.12	3.77±1.34
ΔH_2 / kcal mol^{-1}		-17.2±0.2	-17.8±3.5

Table S5. The sequences of the N30 random domain of the oligonucleotides from the final enriched pool. The prevalence of each oligonucleotide is ranked from highest to lowest based on the number of counts.

No.	Sequences (5' – 3')	Counts (total 50)
1 (SCA2.1)	CTTAAGTGGGGTTCGGGTGGAGTTTATGGG	30
2	TGAGAAGTGTGATTCAGTATGTTTTCCGAA	7
3	CGAGAAGTGTGTTCAGTGAGTTTTCCGAGG	4
4	CGCGGGGGTGGCTGGGGGTGTCTAGCAGAG	2
5	ATTAAGTGGGGTTCGGGTGGAGTTTATGGG	1
6	CCTTGGGTAGGTCAGTGTGGGGTTAGGGA	1
7	CTTAAGTGGGGTTCGGGCGGAGTTTATGGG	1
8	GGGAAGTGGGGTTCGGGTGGTGTTTTCCCA	1
9	GGGATGGGGTGCTCGGTCGGGGGTTGTGAG	1
10	GGTAAGAGTGGTTCCAGTTGAGTTTATGCC	1
11	GGTCAGCACCTGTCGTGGTGGAGGGGTACT	1

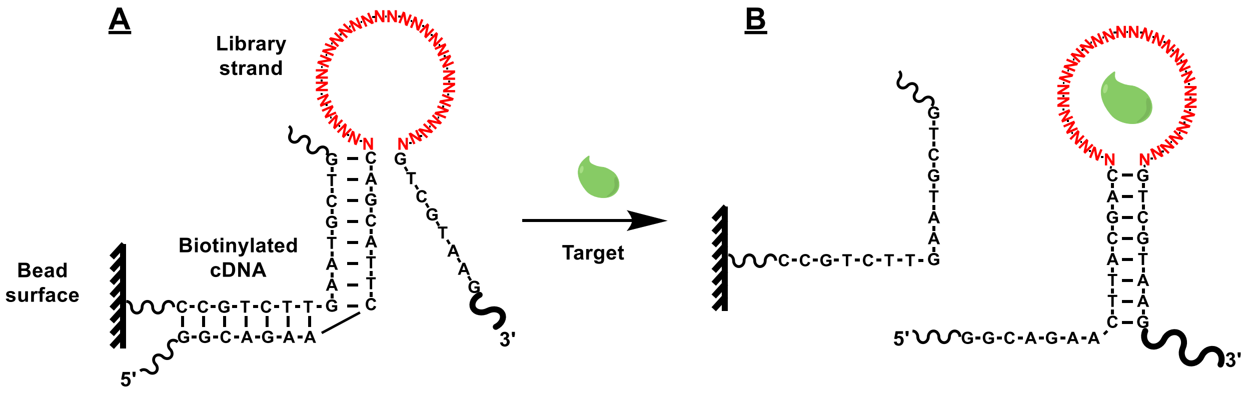

Figure S1. Scheme for library-immobilized SELEX. (**A**) Library molecules are immobilized on streptavidin-coated agarose beads via a biotinylated complementary DNA (cDNA) strand. (**B**) Strands that bind to the target are released into solution via target-induced strand displacement.

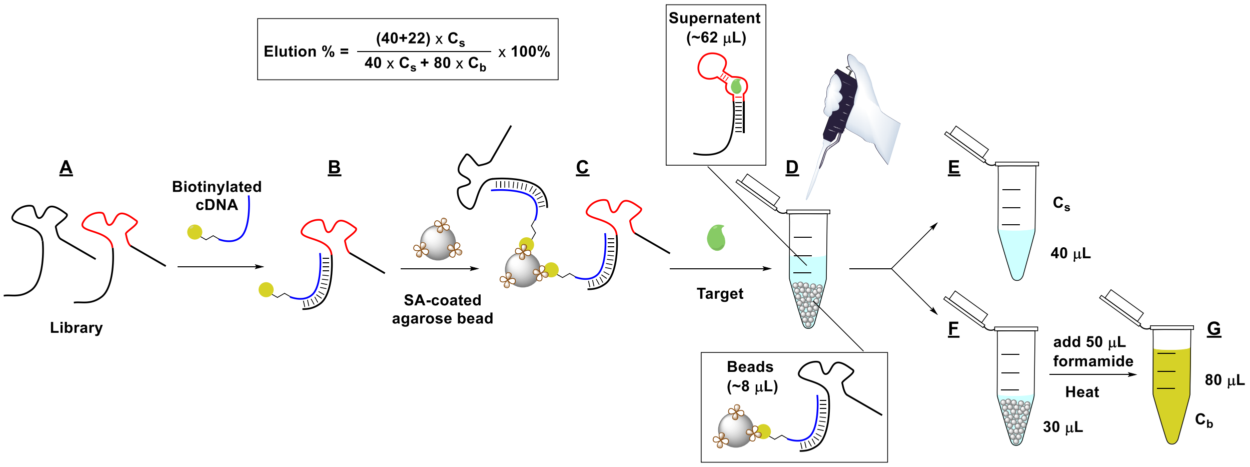

Figure S2. Scheme of the gel elution assay. (**A**) Library strands are hybridized with biotinylated cDNA to form (**B**) library-cDNA complex. (**C**) The resulting complex is immobilized on streptavidin-coated agarose beads. (**D**) The target is added the solution and (**E**) the supernatant containing the target-bound strands is collected and added to a separate tube. (**F**) The leftover beads are treated with formamide-EDTA and heat to (**G**) release remaining non-binding strands from the beads. The quantity of DNA in the solutions obtained from E and G is determined via PAGE; the number of eluted strands is used to calculate the pool binding affinity and specificity using the boxed equation.

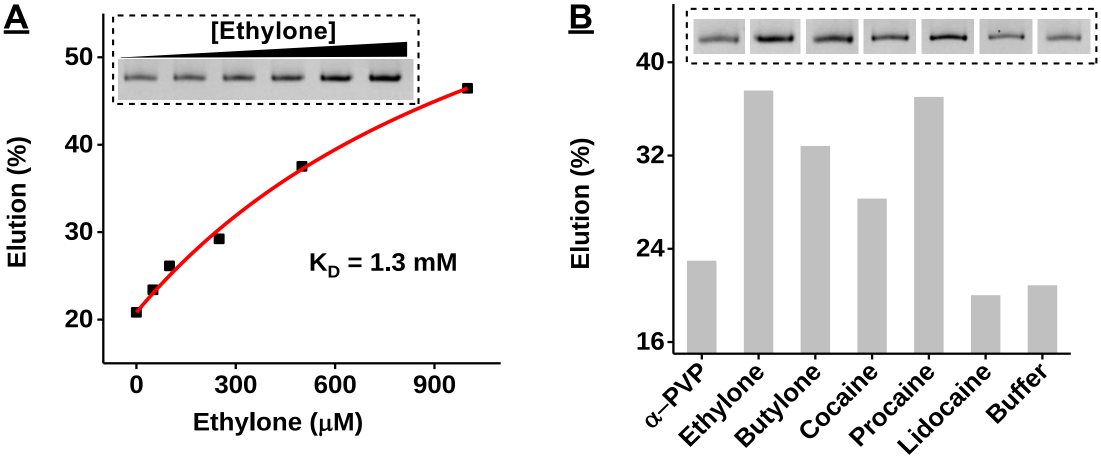

Figure S3. Determination of the target-binding affinity, cross-reactivity, and specificity of the round P5 ethylone pool via gel-elution assay. (**A**) Polyacrylamide gel electrophoresis (PAGE) results depict the target elution profile, with lanes representing samples of the pool eluted with 0, 50, 100, 250, 500, or 1,000 µM ethylone (left to right). The percent of target-eluted pool was plotted against the concentration of ethylone employed for elution to determine the binding affinity of the enriched pool. (**B**) PAGE analysis (top) of elution with 500 µM synthetic cathinones (α-PVP, ethylone, butylone) or interferents (cocaine, procaine, lidocaine) was used to measure cross-reactivity and specificity of the enriched P5 pool.

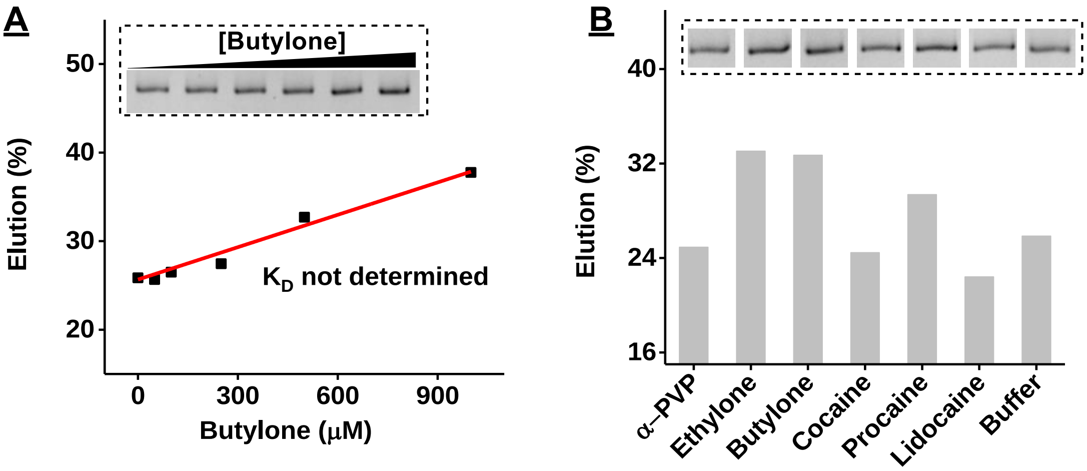

Figure S4. Determination of the target-binding affinity, cross-reactivity, and specificity of the round P5 butylone pool via gel-elution assay. (**A**) PAGE results depict the target elution profile, with lanes representing samples of the pool eluted with 0, 50, 100, 250, 500, or 1,000 μM butylone (left to right). The percent of target-eluted pool was plotted against the concentration of butylone employed for elution to determine the binding affinity of the enriched pool. (**B**) PAGE analysis (top) of elution with 500 μM synthetic cathinones (α-PVP, ethylone, butylone) or interferents (cocaine, procaine, lidocaine) was used to measure cross-reactivity and specificity of the enriched P5 pool.

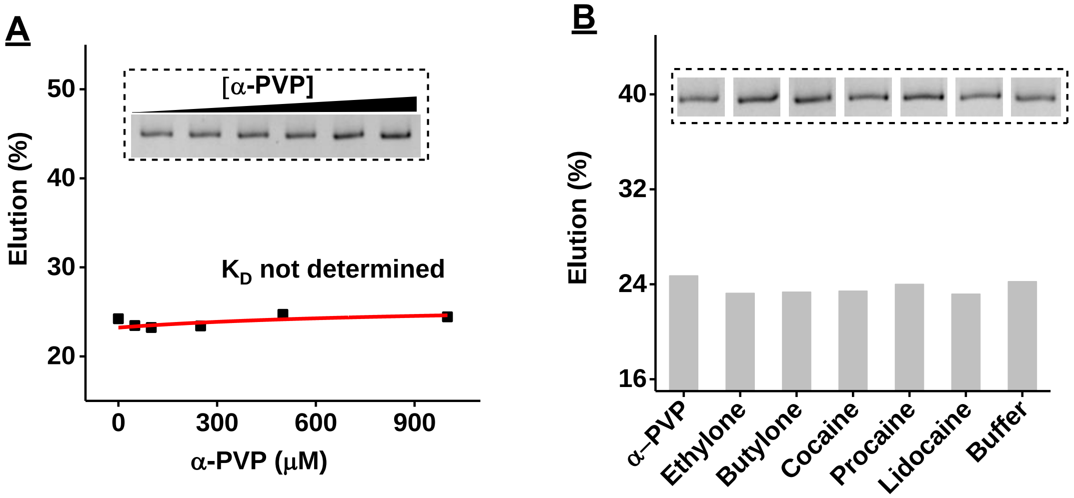

Figure S5. Determination of the target-binding affinity, cross-reactivity, and specificity of the round P5 α-PVP pool via gel-elution assay. (**A**) PAGE results depict the target elution profile, with lanes representing samples of the pool eluted with 0, 50, 100, 250, 500, or 1,000 μM α-PVP (left to right). The percent of target-eluted pool was plotted against the concentration of α-PVP employed for elution to determine the binding affinity of the enriched pool. (**B**) PAGE analysis (top) of elution with 500 μM synthetic cathinones (α-PVP, ethylone, butylone) or interferents (cocaine, procaine, lidocaine) was used to measure target-cross-reactivity and specificity of the enriched P5 pool.

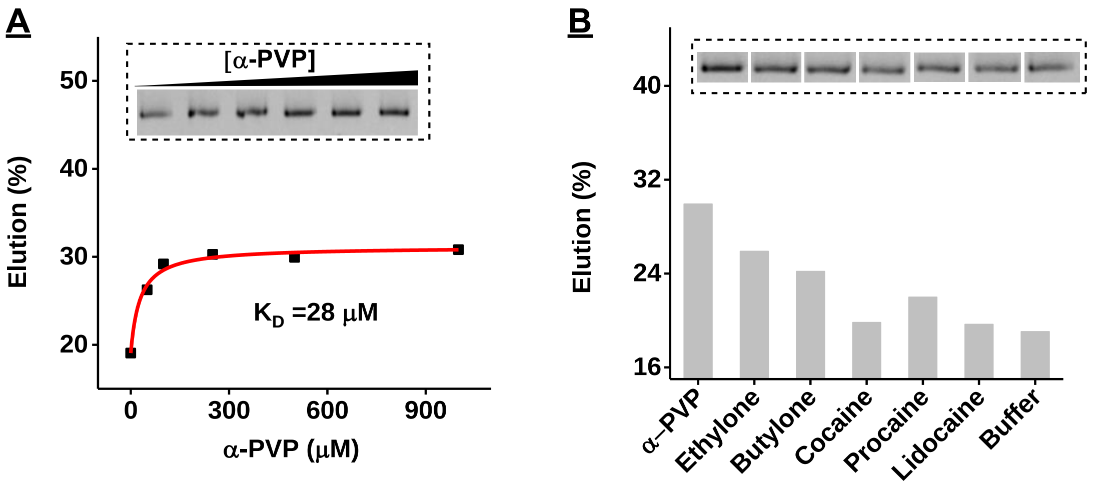

Figure S6. Determination of the target-binding affinity, cross-reactivity, and specificity of the round P9 α-PVP pool via gel-elution assay. (**A**) PAGE results depict the target elution profile, with lanes representing samples of the pool eluted with 0, 50, 100, 250, 500, or 1,000 μM α-PVP (left to right). The percent of target-eluted pool was plotted against the concentration of α-PVP employed for elution to determine the binding affinity of the enriched pool. (**B**) PAGE analysis (top) of elution with 500 μM synthetic cathinones (α-PVP, ethylone, butylone) or interferents (cocaine, procaine, lidocaine) was used to measure target-cross-reactivity and specificity of the enriched P9 pool.

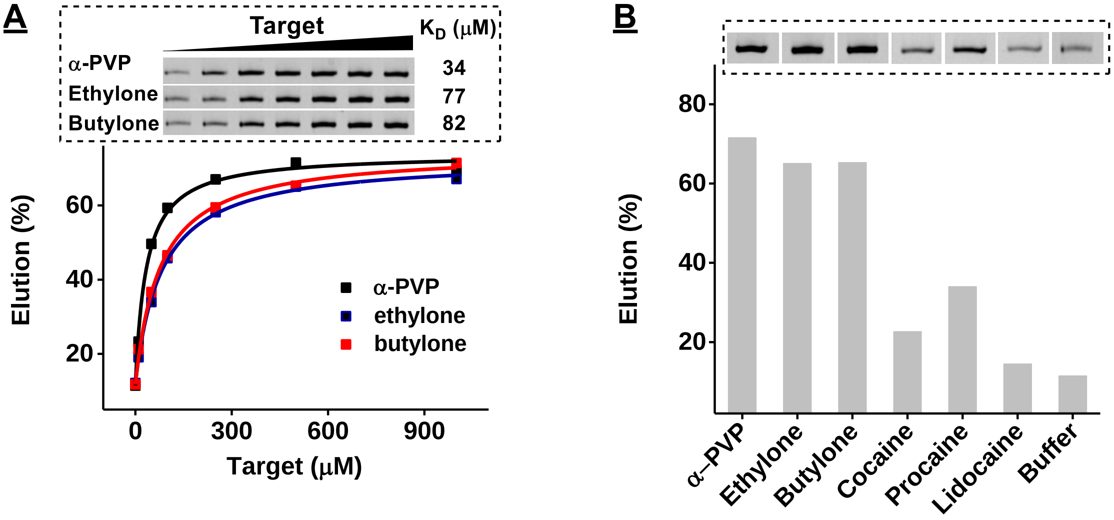

Figure S7. Determination of the target-binding affinity, cross-reactivity and specificity of the round S3 pool via a gel elution assay. (**A**) PAGE results depict the target elution profile with lanes representing samples of the pool eluted with 0, 10 50, 100, 250, 500, or 1,000 μM (left to right) of α-PVP, ethylone, or butylone. The percent of target-eluted pool was plotted against the concentration of target used for elution to determine the binding affinity of the enriched pool. (**B**) PAGE analysis of enriched pool eluted with 500 μM synthetic cathinones (α-PVP, ethylone, butylone) or interferents (cocaine, procaine, lidocaine) was used to measure target-cross-reactivity and specificity of the enriched S3 pool.

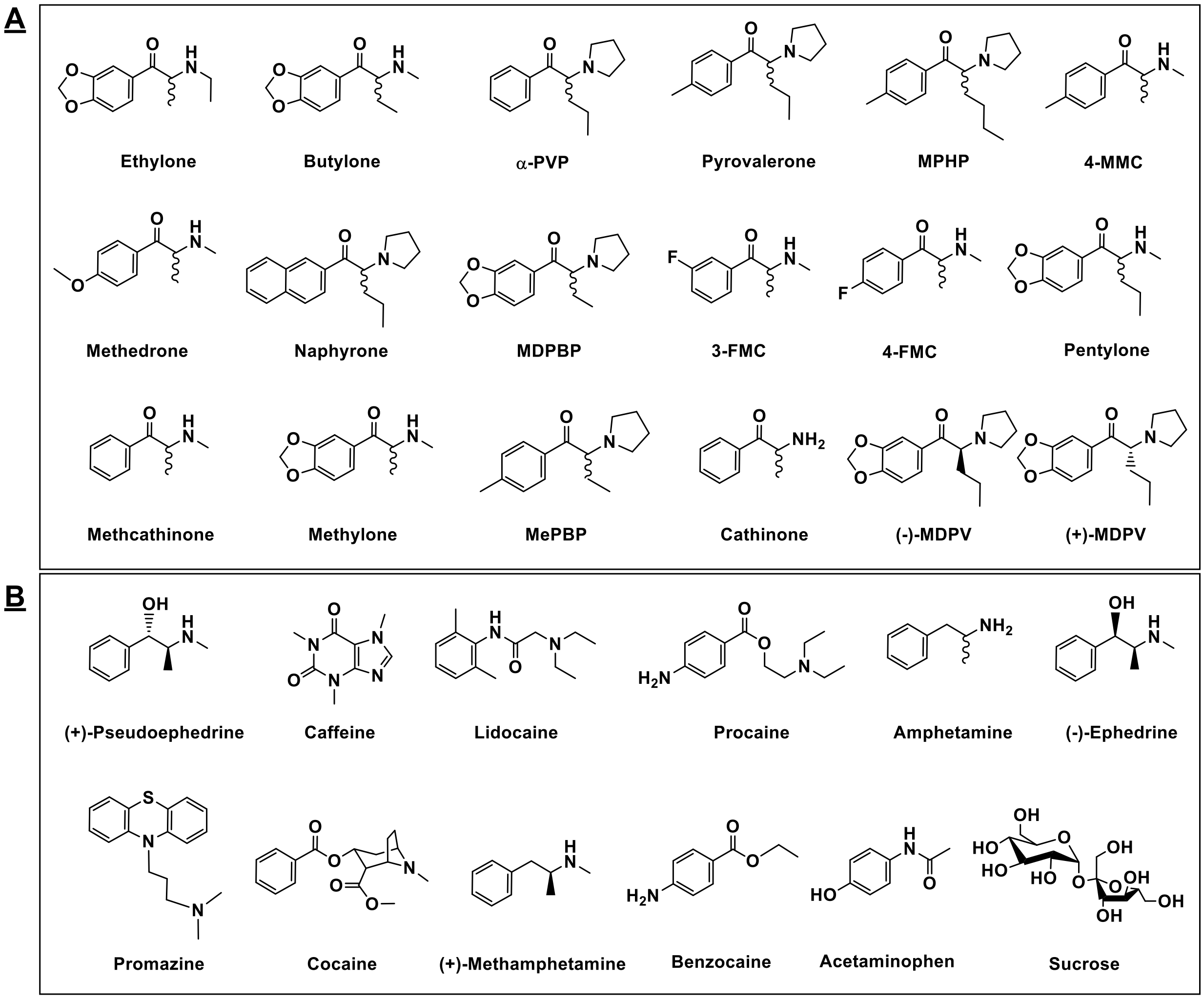

Figure S8. The chemical structures of the (**A**) synthetic cathinones and (**B**) interferent compounds used in this work. Note that racemic MDPV was also used in this work.

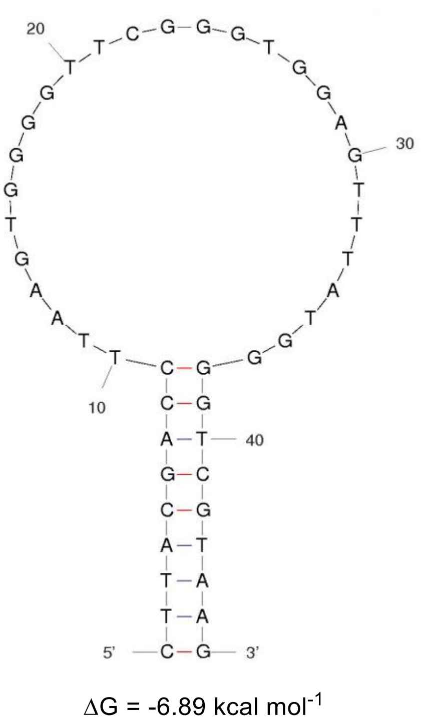

Figure S9. Secondary structure of SCA2.1 as predicted by Mfold at selection buffer ion concentrations (20 mM Na^+ and 0.5 mM Mg^{2+}) at 23 ºC. Estimated free energy of formation (ΔG) is also shown.

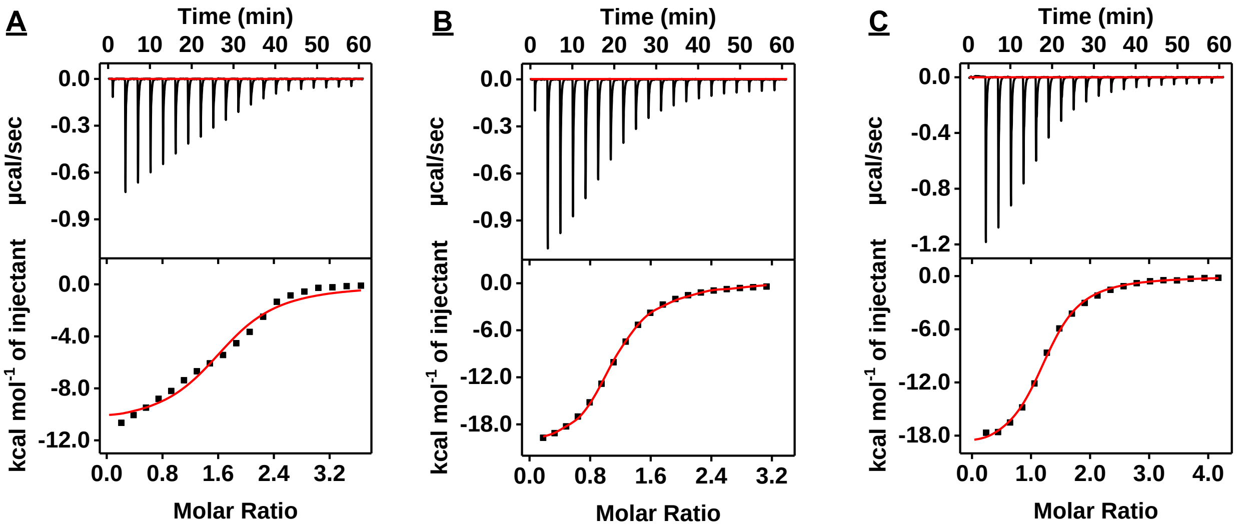

Figure S10. Characterization of the target-binding affinity of SCA2.1 using isothermal titration calorimetry (ITC). Top panels present raw data showing the heat generated from each titration of (**A**) α-PVP, (**B**) ethylone and (**C**) butylone to SCA2.1, while bottom panels show the integrated heat of each titration after correcting for dilution heat of the titrant. ITC data were fitted using a single-site model and the binding parameters are shown in Table S4.

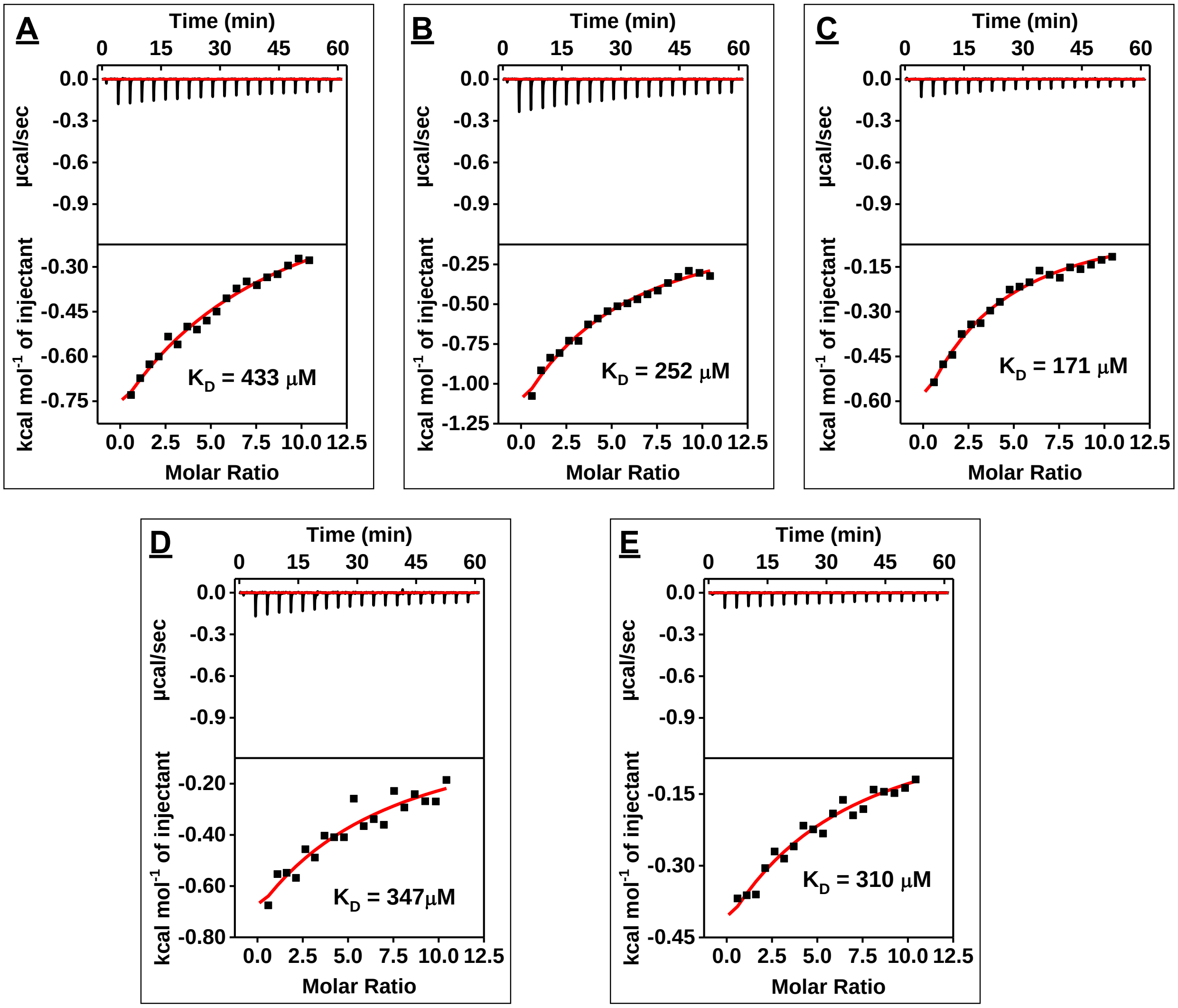

Figure S11. Characterization of the interferent-binding affinity of SCA2.1 using ITC. Top panels present raw data showing the heat generated from each titration of (**A**) amphetamine, (**B**) methamphetamine, (**C**) ephedrine, (**D**) cocaine, and (**E**) procaine to SCA2.1, while bottom panels show the integrated heat of each titration after correcting for dilution heat of the titrant. ITC data were fitted with a one-site binding model and K_Ds are shown in the figures.

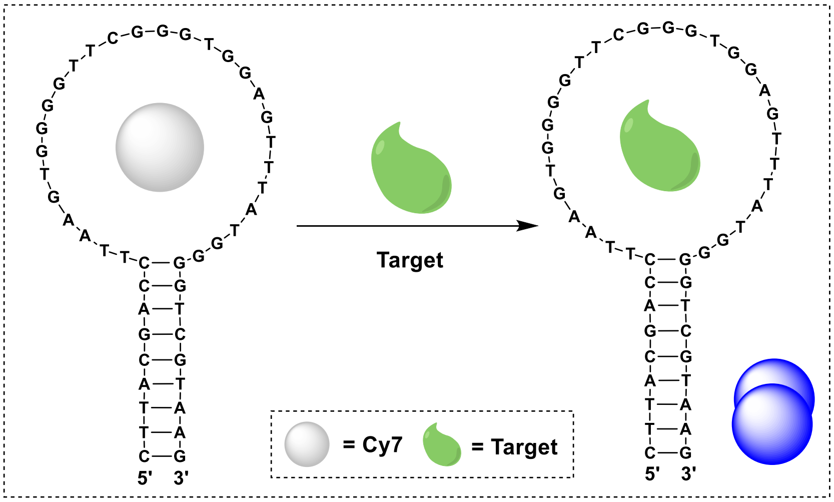

Figure S12. Colorimetric detection of synthetic cathinones using a Cy7-displacement assay. Schematic of the Cy7-displacement assay, wherein binding of a synthetic cathinone molecule displaces the Cy7 monomer from the binding domain of SCA2.1, inducing formation of Cy7 dimers that produce a change in the absorbance of the dye.

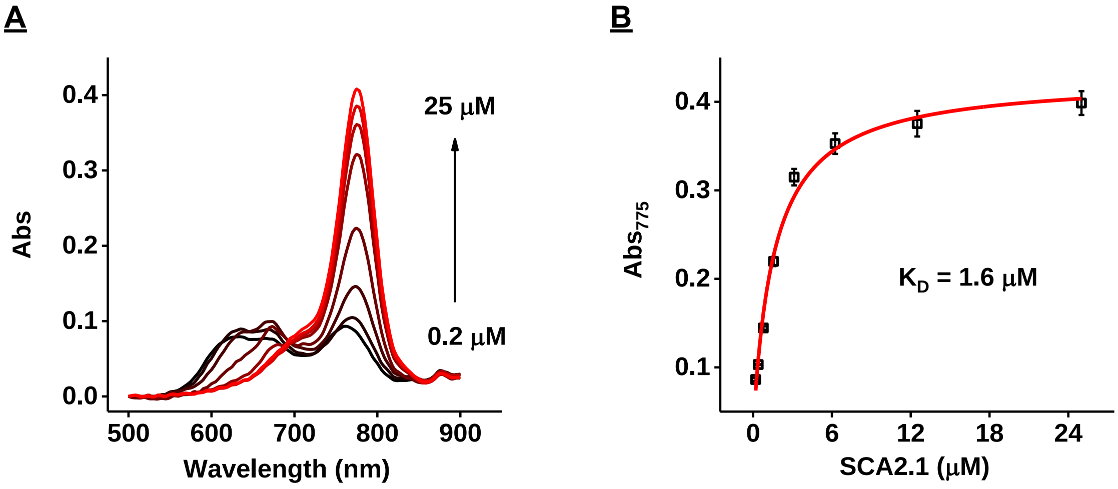

Figure S13. Determination of the binding affinity of Cy7 to SCA2.1 via a colorimetric assay. (**A**) Absorbance spectra of 2 µM Cy7 in the presence of 0.2, 0.4, 0.8, 1.6, 3.1, 6.3, 12.5, or 25 µM SCA2.1, with the black-to-red color gradient representing increasing concentrations. (**B**) Plot of the absorbance of Cy7 monomer at its peak wavelength (775 nm) versus concentration of SCA2.1, fitted with the Langmuir equation to determine the binding affinity of Cy7 to SCA2.1.

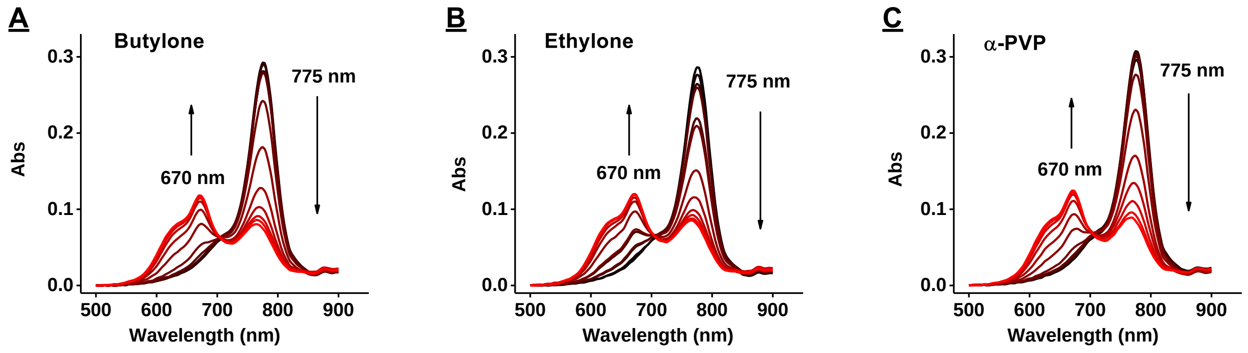

Figure S14. Cy7-displacement colorimetric assay for the detection of synthetic cathinones using SCA2.1. Absorbance spectra of Cy7 (2 μM) in the presence of 0, 0.1, 0.3, 0.5, 1.1, 2.3, 4.9, 10.3, 21.6, 45.4, 95.2, or 200 μM (**A**) butylone, (**B**) ethylone, (**C**) α-PVP, with the black-to-red color gradient representing increasing concentrations of target. [SCA2.1] = 3 μM.

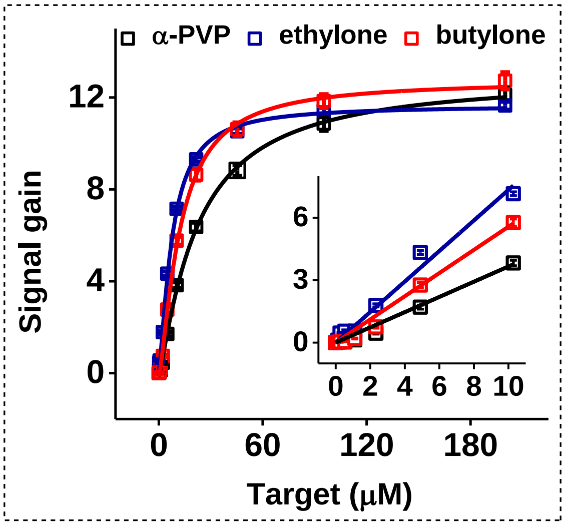

Figure S15. Calibration curves based on the absorbance ratio at 670/775 nm in the presence of different concentrations of α-PVP, ethylone or butylone in buffer. The inset represents the linear range at 0 to 10 μM target. Error bars show standard deviation from three measurements at each concentration. [SCA2.1] = 3 μM, [Cy7] = 2 μM.

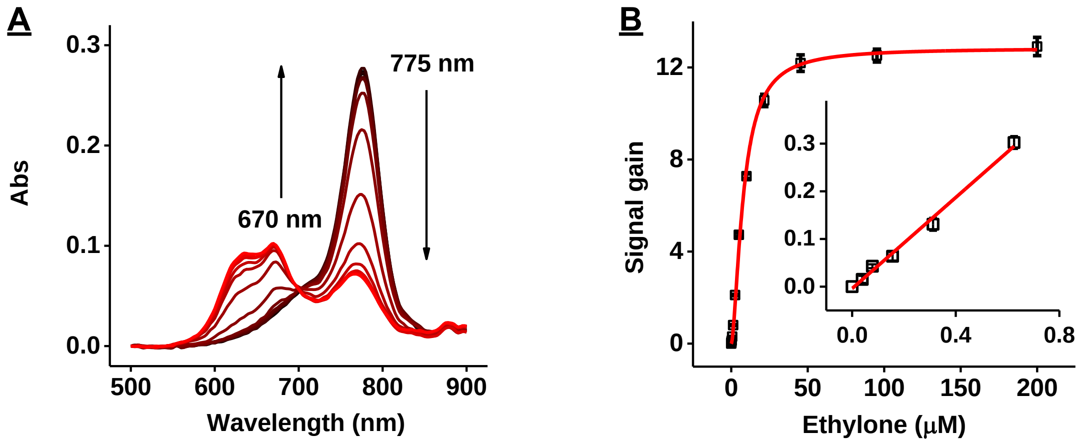

Figure S16. Detection of ethylone in 50% urine using an SCA2.1-based Cy7-displacement colorimetric assay. (**A**) Absorbance spectra of Cy7 (2 μM) in the presence of 0, 0.03, 0.06, 0.12, 0.25, 0.53, 1.11, 2.33, 4.90, 10.28, 21.60, 45.35, 95.24, or 200 μM ethylone, with the black-to-red color gradient representing increasing concentrations of target. (**B**) Assay calibration curve generated using 0-200 μM ethylone, with the inset representing the range of target concentrations from 0 to 1.1 μM. [SCA2.1] = 3 μM.

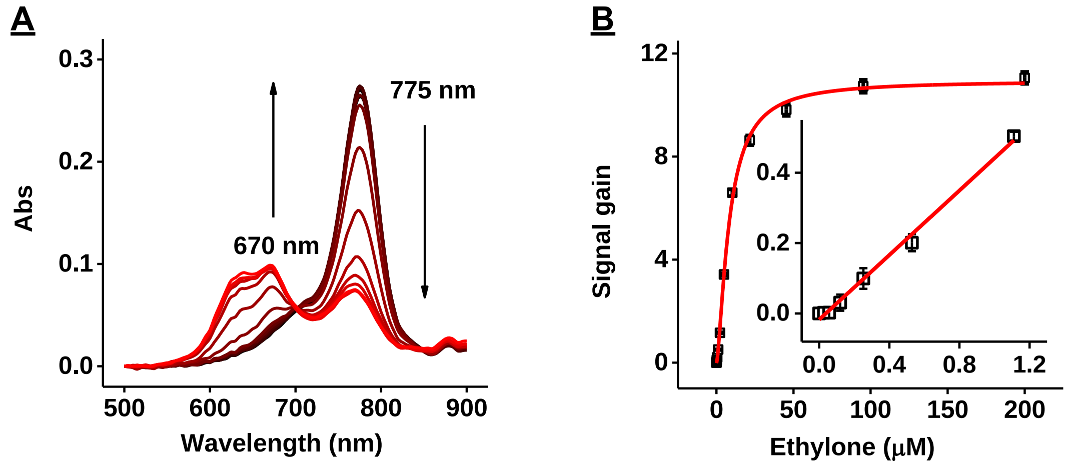

Figure S17. Detection of ethylone in 50% saliva using the SCA2.1-based Cy7-displacement colorimetric assay. (**A**) Absorbance spectra of Cy7 (2 μM) in the presence of 0, 0.03, 0.06, 0.12, 0.25, 0.53, 1.11, 2.33, 4.90, 10.28, 21.60, 45.35, 95.24, or 200 μM ethylone, with the black-to-red color gradient representing increasing concentrations of target. (**B**) Assay calibration curve generated using 0-200 μM ethylone, with the inset representing the range of target concentrations from 0 to 1.1 μM. [SCA2.1] = 3 μM.

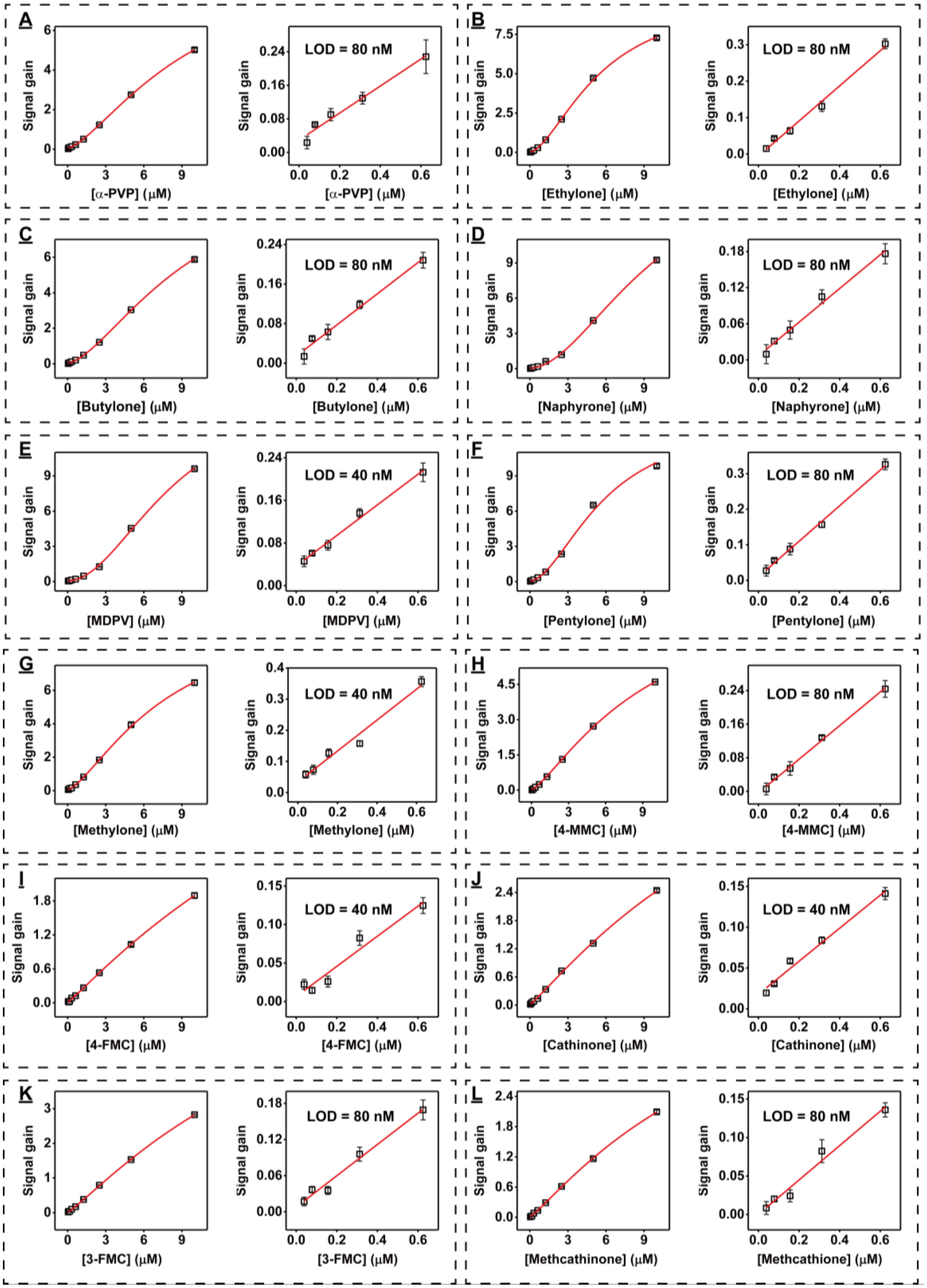

Figure S18. Calibration curves and linear ranges for the SCA2.1-based Cy7-displcaement assay in 50% urine based on the absorbance ratio at 670/775 nm in the presence of different concentrations of various synthetic cathinones. Error bars show standard deviation from three measurements at each concentration. [SCA2.1] = 3 μM, [Cy7] = 2 μM.

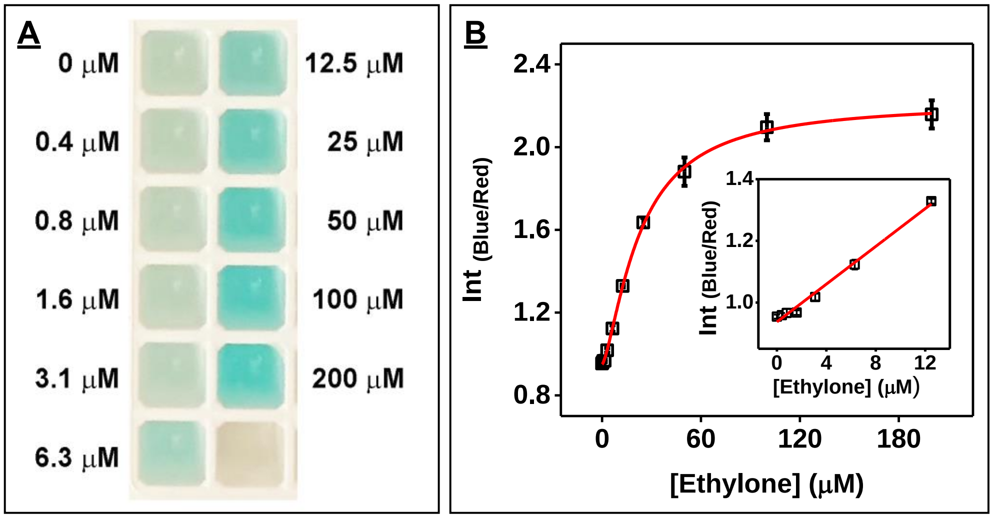

Figure S19. Naked-eye detection of ethylone in the concentration range of 0.4 μM to 200 μM. (**A**) Photograph of the assay. The blue color change can be clearly observed at concentrations ≥6.3 μM target. (**B**) A calibration curve based on the photograph was made using Image-J software. [Cy7] = 3.5 μM. [SCA2.1] = 5 μM.
